# Wet-dry-wet drug screen leads to the synthesis of TS1, a novel compound reversing lung fibrosis through inhibition of myofibroblast differentiation

**DOI:** 10.1038/s41419-021-04439-4

**Published:** 2021-12-17

**Authors:** Nadja Anneliese Ruth Ring, Maria Concetta Volpe, Tomaž Stepišnik, Maria Grazia Mamolo, Panče Panov, Dragi Kocev, Simone Vodret, Sara Fortuna, Antonella Calabretti, Michael Rehman, Andrea Colliva, Pietro Marchesan, Luca Camparini, Thomas Marcuzzo, Rossana Bussani, Sara Scarabellotto, Marco Confalonieri, Tho X. Pham, Giovanni Ligresti, Nunzia Caporarello, Francesco S. Loffredo, Daniele Zampieri, Sašo Džeroski, Serena Zacchigna

**Affiliations:** 1 Cardiovascular Biology Laboratory, International Centre for Genetic Engineering and Biotechnology (ICGEB), Trieste, Italy; 2 Department of Medical, Surgical and Health Sciences, University of Trieste, Trieste, Italy; 3 Jožef Stefan Institute, Ljubljana, Slovenia; Jožef Stefan International Postgraduate School, Ljubljana, Slovenia; 4 Department of Chemical and Pharmaceutical Sciences, University of Trieste, Trieste, Italy; 5 Yale School of Medicine Section of Nephrology, New Haven, CT USA; 6 Molecular Cardiology Unit, International Centre for Genetic Engineering and Biotechnology (ICGEB), Trieste, Italy; 7 Arthritis Center, Department of Medicine, Boston University School of Medicine, Boston, MA USA; 8 Department of Physiology and Biomedical Engineering, Mayo Clinic, Rochester, MN USA; 9 Department of Translational Medical Sciences, University of Campania “Luigi Vanvitelli”, Naples, Italy

**Keywords:** Drug discovery, Respiratory tract diseases

## Abstract

Therapies halting the progression of fibrosis are ineffective and limited. Activated myofibroblasts are emerging as important targets in the progression of fibrotic diseases. Previously, we performed a high-throughput screen on lung fibroblasts and subsequently demonstrated that the inhibition of myofibroblast activation is able to prevent lung fibrosis in bleomycin-treated mice. High-throughput screens are an ideal method of repurposing drugs, yet they contain an intrinsic limitation, which is the size of the library itself. Here, we exploited the data from our “wet” screen and used “dry” machine learning analysis to virtually screen millions of compounds, identifying novel anti-fibrotic hits which target myofibroblast differentiation, many of which were structurally related to dopamine. We synthesized and validated several compounds ex vivo (“wet”) and confirmed that both dopamine and its derivative TS1 are powerful inhibitors of myofibroblast activation. We further used RNAi-mediated knock-down and demonstrated that both molecules act through the dopamine receptor 3 and exert their anti-fibrotic effect by inhibiting the canonical transforming growth factor β pathway. Furthermore, molecular modelling confirmed the capability of TS1 to bind both human and mouse dopamine receptor 3. The anti-fibrotic effect on human cells was confirmed using primary fibroblasts from idiopathic pulmonary fibrosis patients. Finally, TS1 prevented and reversed disease progression in a murine model of lung fibrosis. Both our interdisciplinary approach and our novel compound TS1 are promising tools for understanding and combating lung fibrosis.

## Introduction

Fibrosis-related mortality is on the rise. Mortality data from the world health organization between 2001 and 2013 shows a general trend of increasing mortality for lung fibrosis in the EU [1]. This can be explained through several factors, including an aging population and insufficient treatment options to prevent or cure these disorders, due to the large diversity in both aetiology and symptoms [2].

Inflammatory cells are activated in response to tissue damage, leading to the proliferation of fibroblasts and their differentiation into activated myofibroblasts, as an attempt to repair the injury. In a later phase, inflammation decreases, and a scar is formed [3]. A major inducer of myofibroblast activation is transforming growth factor β (TGFβ) [4], which increases the secretion of extracellular matrix components, and the expression of the contractile protein alpha-smooth muscle actin (αSMA), currently considered to be the most reliable marker of this cell type [5].

While many therapeutic approaches focus on the inflammatory component of fibrosis, the use of corticosteroids as anti-inflammatory therapy has been shown to be ineffective and fraught with side effects [6, 7]. Pirfenidone and nintedanib are two drugs recently approved for the treatment of lung fibrosis, and while their exact mechanism of action has not been described, part of their anti-fibrotic activity is through suppression of inflammation [8]. Aside from inflammation, the key role of activated myofibroblasts in the development of fibrotic tissue is emerging as a crucial factor [5]. One therapeutic approach targeting these cells is the development of Bcl-2 homology domain mimetics, which target the anti-apoptotic Bcl2 protein and induce the apoptosis of myofibroblasts, bringing with it obvious risks and side effects [9].

While myofibroblasts have been the target of therapies, few approaches have attempted to interfere with fibroblast differentiation into activated myofibroblasts [10]. In a previous study of our laboratory, we performed a high-throughput screen using a library of FDA-approved chemical compounds and observed their effect on ex vivo lung fibroblasts. Using the top hits of this screen we demonstrated that the inhibition of myofibroblast activation, shown by the accumulation of the contractile protein αSMA is able to prevent the development of lung fibrosis in a murine model of lung fibrosis [11].

Here, we apply an interdisciplinary wet-dry-wet approach. We exploited data generated in our previously performed “wet” high-throughput screen, and used “dry” machine learning to virtually screen the ChEMBL database. ChEMBL is a publicly available database comprising a large collection of drug-like small molecules, including information about their structure, properties, and bioactivity [12, 13]. This enabled us to overcome the intrinsic limit of the compound library size in the original screen and extrapolate potential hits among millions of compounds, thereby increasing both coverage and value of the screen. Finally, we validated the predicted anti-fibrotic compounds in both primary cells and a mouse model of lung fibrosis (“wet”). Using this wet-dry-wet approach, we selected and synthesized several compounds and identified TS1 as the most promising anti-fibrotic agent.

## Results

### Machine learning analysis of a previous drug screen identifies novel anti-fibrotic candidates

We recently performed a high content high throughput screen (HC-HTS), using a library of 640 FDA-approved compounds [11]. The screen used primary fibroblasts from αSMA-RFP/COLL-EGFP mice [14], which express red fluorescent protein (RFP) under the alpha smooth muscle actin (αSMA) promoter and enhanced green fluorescent protein (EGFP) under the collagen α1(I) promoter. These cells differentiate into myofibroblasts in culture, either spontaneously or upon exposure to transforming growth factor beta (TGFβ). This screen led to the identification of haloperidol as an inhibitor of myofibroblast activation and a potent anti-fibrotic agent in vivo.

To maximize the quality and the coverage of our “wet” screen in primary fibroblasts, we applied a machine learning approach to its results and learned a model to predict the anti-fibrotic effect of compounds not included in the original screen. Machine learning algorithms have the ability to learn and improve with experience presented in the form of data. Data consist of examples that typically reside in a table, where each row represents an example: in our case, each row represents a chemical compound. Each column from the table represents a specific property or descriptor of the compounds, in this case, extended connectivity fingerprints (ECFP). ECFPs are often used and typically prove to be well suited for predicting biological properties of molecules. They describe compounds with binary vectors, and each vector component corresponds to a set of substructures. If that component has a value of 1, this means that at least one of those substructures is present in the molecule. The dataset used to learn the models is usually called the training set, and the output is a predictive algorithm that can then be applied to the so-called testing set.

In this study, we used our existing “wet” screening data, i.e., experimental measurements of average RFP/EGFP intensity, as our training set. The predictive model has the form of a bagging ensemble of predictive clustering trees (Kocev, Vens et al. 2013), which simultaneously predict the effect of a compound on both RFP and EGFP intensity. Performance of the model was measured by mean absolute error, with performance on unseen compounds estimated via 10-fold cross-validation. The mean absolute errors of the model were estimated to be 0.0836 for EGFP and 0.0871 for RFP.

To discover novel compounds able to lower αSMA expression, and thus, presumably, myofibroblast activation, we used the learned model to virtually screen our testing set: the ChEMBL database (containing over 1.7 million compounds in total). For each compound, its ECFP was obtained and fed to the predictive model to obtain a prediction of the compound effect on RFP/EGFP intensity. We performed a subsequent filtering step to filter the weak predictions and make a priority list for further testing and examination.

**Fig. 1:**
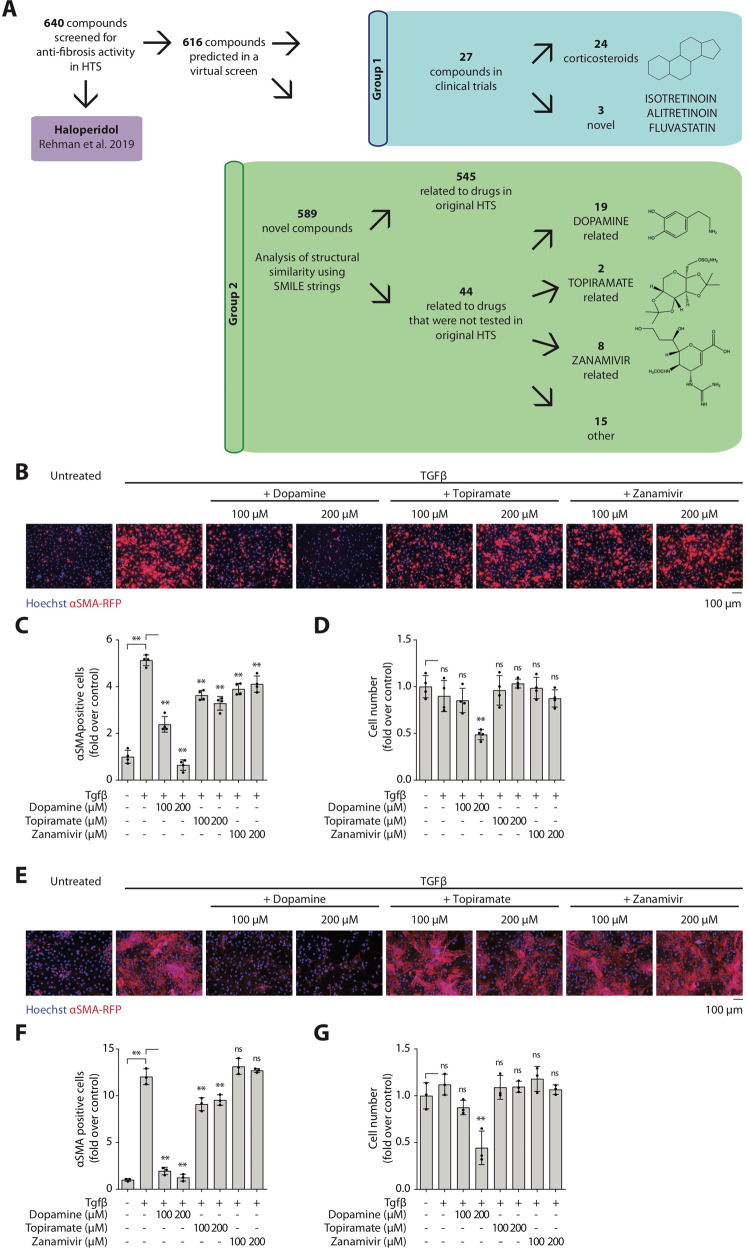
Virtual screen identifies dopamine as a powerful anti-fibrotic agent in primary murine fibroblasts. **A** Flowchart showing the data processing of the machine learning predictions. In a previous publication, 600 compounds were screened, identifying haloperidol as a potent anti-fibrotic drug (purple). In the virtual screen, 616 compounds are predicted to decrease αSMA expression by 25% and are divided into two groups. Group 1 (blue) contains all predicted compounds which are clinically approved. Group 2 (green) contains unapproved, novel, predicted compounds. These were further analyzed to identify structurally related, clinically approved, drugs. Of these, 545 compounds were structurally related to drugs present in the original “wet” screen, while the remaining 44 drugs were related to dopamine (19/44), topiramate (2/44), zanamivir (8/44), and others (15/44). **B** Murine heart fibroblasts were treated ex vivo with TGFβ and dopamine, topiramate, and zanamivir. Representative images are shown (Hoechst in blue, αSMA-RFP in red). **C** Quantification of the number of αSMA positive cells (shown as fold over control). **D** Quantification of the total number of cells per condition (shown as fold over control). **E** Murine lung fibroblasts were treated ex vivo with TGFβ and dopamine, topiramate, and zanamivir. Representative images are shown (Hoechst in blue, αSMA-RFP in red). **F** Quantification of the number of αSMA positive cells (shown as fold over control). **G** Quantification of the total number of cells per condition (shown as fold over control). All data are shown as mean ± SD. Statistical significance was determined using a one-way ANOVA followed by Dunnett’s multiple comparison test, *P < 0.05, **P < 0.01, *n* = 3.

This algorithm selected 616 candidates, which were predicted to reduce αSMA intensity by at least 25% compared to controls (Fig. 1A). After thresholding, compounds present in the original screen were removed from the candidate list. The remaining compounds were divided into two groups. Group 1 contained compounds that have already reached clinical trial stage, while novel compounds were included in Group 2. Group 1 was composed of 27 compounds, of which 24 were corticosteroids. Based on their potent anti-inflammatory activity, corticosteroids have been extensively considered for use as anti-fibrotic drugs [15–18]. However, they have invariably failed, exhibiting severe side effects [6, 7, 19] and therefore we did not consider them further. The three remaining compounds in group 1 were isotretinoin, alitretinoin, and fluvastatin, which belong to well-known classes of drugs and ranked relatively high in our original HTS, namely retinoids and statins. On the one hand, this renders them of little interest for our purpose, while on the other it confirms the power of our virtual screen in identifying novel therapeutic candidates.

Thus, we focused on Group 2, made up of 589 novel compounds. We used simplified molecular-input line-entry system (SMILES) in the online tool ChemMine to group them according to their structural similarity to known drugs [20]. The majority (*n* = 545) contained structural components of FDA-approved drugs which were contained in our original screen compound library. Specifically, 327 compounds were related to the corticosteroid dexamethasone, 219 were related to either amiodarone, celecoxib, chlorambucil, ciprofloxacin, indomethacin, losartan, melatonin, or paclitaxel. Interestingly, the remaining 44 compounds were related to drugs unrepresented in the original HTS, including acyclovir, prasterone, progesterone, thiamine ion, topiramate, zanamivir, and dopamine. In particular, structural components of dopamine were contained in 43% of these compounds. In total, three candidates were chosen for validation in vitro, dopamine, topiramate, and zanamivir, as they have never been specifically considered in relation to fibrotic disorders.

### Dopamine inhibits myofibroblast activation

To validate the efficacy of the selected drugs in reducing αSMA expression, primary murine cardiac fibroblasts were isolated from αSMA-RFP mice, as in our original screen. Cells were stimulated for 48 h with TGFβ and treated with dopamine, topiramate, or zanamivir [21, 22]. At 100 µM dopamine was clearly the most effective one in reducing αSMA expression (Fig. 1B, C). The effect was more evident at 200 µM, but this dose resulted in reduced cell viability (Fig. 1D).

Given the current need for new therapeutics for lung fibrosis, we tested whether a similar effect was exerted in primary lung fibroblasts from αSMA-RFP mice and confirmed the results (Fig. 1E–G). In this case, the potent anti-fibrotic effect of dopamine was even more evident at 100 µM. These results confirm the ability of our virtual screen to identify interesting leads and justify further in-depth investigation on the mechanism of action of dopamine.

### Dopamine reduces myofibroblast activation through the dopamine receptor 3

**Fig. 2:**
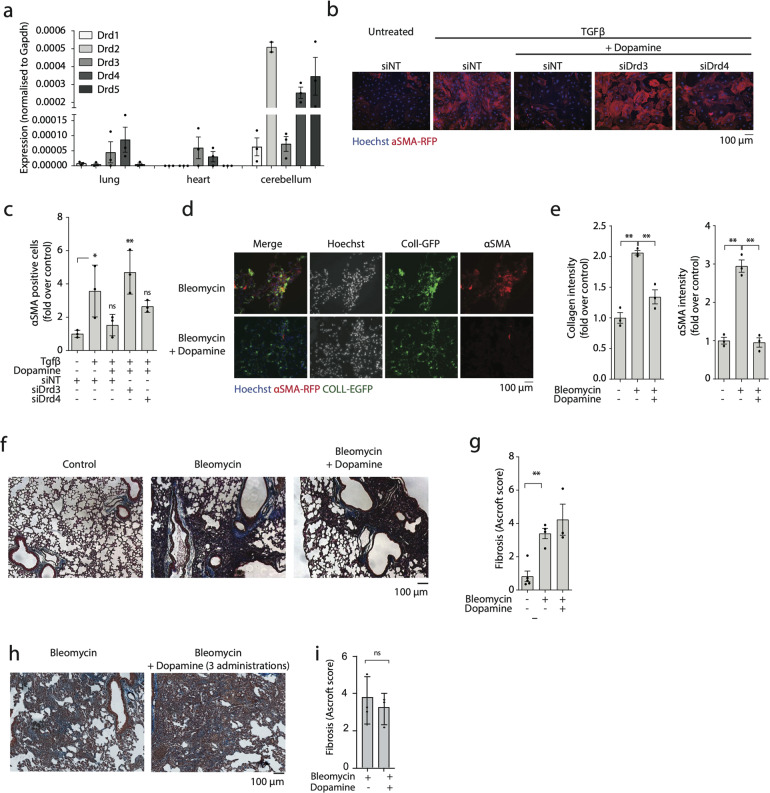
Dopamine inhibits lung myofibroblast differentiation by acting through Drd3 but is unable to prevent bleomycin-induced lung fibrosis in vivo. **a** RT-PCR analysis shows the abundance of each dopamine receptor in fibroblasts from the murine lung and heart, using cerebellum lysate as a positive control. Results are normalized to *Gapdh* expression. **b** Murine lung fibroblasts were treated ex vivo with non-targeting siRNA (siNT) or pools of two siRNAs targeting either Drd3 or Drd4. After 48 h, cells were treated with TGFβ and dopamine for an additional 48 h prior to fixation. Representative images are shown (Hoechst in blue, αSMA-RFP in red). **c** Quantification of the number of αSMA positive cells (shown as fold over control). **d** COLL-EGFP mice were treated with bleomycin and dopamine by intratracheal injection and lungs were harvested after 14 days. Representative images are shown, with nuclei shown in blue, COLL-EGFP in green, and αSMA in red. **e** Quantification of collagen and αSMA intensity of lungs shown in panel (**d**), normalized over untreated control lungs. **f** Mice were treated with bleomycin, either alone or in combination with dopamine, by intratracheal injection. Lungs were harvested after 30 days and stained with Masson-Trichrome to visualize collagen fibres in blue. **g** Quantification of lung fibrosis using the Ashcroft score. **h** Mice were treated with bleomycin by intratracheal injection. Dopamine was administered at 7, 14, and 21 days after bleomycin injection. Lungs were harvested after 30 days and stained with Masson-Trichrome to visualise collagen fibres in blue. **i** Quantification of lung fibrosis using the Ashcroft score. Data in panels **a**, **e**, **g**, and **i** are shown as mean ± S.E.M., while data in panel **c** is shown as ± SD. Statistical significance was determined using a one-way ANOVA followed by Dunnett’s multiple comparison test, *P < 0.05, **P < 0.01.

In order to elucidate the receptor through which dopamine is able to counteract fibroblast differentiation into myofibroblasts, we tested the expression levels of the various dopamine receptors (Drd) in primary fibroblasts from mouse heart and lung, using cerebellum as a positive control. We found that among the Drd family members, Drd3 and Drd4 were primarily expressed by both types of fibroblasts (Fig. 2a). To assess their functional relevance in mediating the anti-fibrotic effect of dopamine, we silenced either receptor using specific siRNAs. The knock down of Drd3 (efficacy shown in Supplementary Fig. 1) significantly inhibited the effect of dopamine (Fig. 2b, c), pointing to this receptor as the major mediator of dopamine activity.

In fact, when we used Western blotting to confirm the protein expression of Drd3 and Drd4 we found that while both are found at detectable levels in brain and cerebellum extracts, only the former can be detected in primary murine lung fibroblasts (Supplementary Fig. 2). This strengthens the evidence that dopamine acts through Drd3, as it is expressed on both RNA and protein level.

We then tested our compounds using the bleomycin model of lung fibrosis applied to the COLL-EGFP transgenic mice. Animals were treated with dopamine through intra-tracheal delivery two days after bleomycin administration and sacrificed at two weeks. As shown in Fig. 2d, e, bleomycin injection resulted in strong up-regulation of collagen expression by lung fibroblasts, together with expression of αSMA in the same cells. Dopamine treatment significantly blunted this response, reducing the levels of both fibrotic markers, especially αSMA, in lung tissue. However, reduced myofibroblast differentiation in long term dopamine-treated lungs did not significantly alter the development of fibrosis, as assessed by Masson Trichrome staining and quantified using the Ashcroft score (Fig. 2f, g). This was not improved even when dopamine was administered repeatedly, at 7, 14, and 21 days following bleomycin administration (Fig. 2h, i).

Collectively, these results indicate that dopamine effectively reduces myofibroblast differentiation ex vivo through Drd3. However, it fails to significantly inhibit bleomycin-induced lung fibrosis in vivo, possibly because of residual collagen expression and/or its quick degradation [23].

### A novel, dopamine-like derivative inhibits the activation of lung myofibroblasts via Drd3

**Fig. 3:**
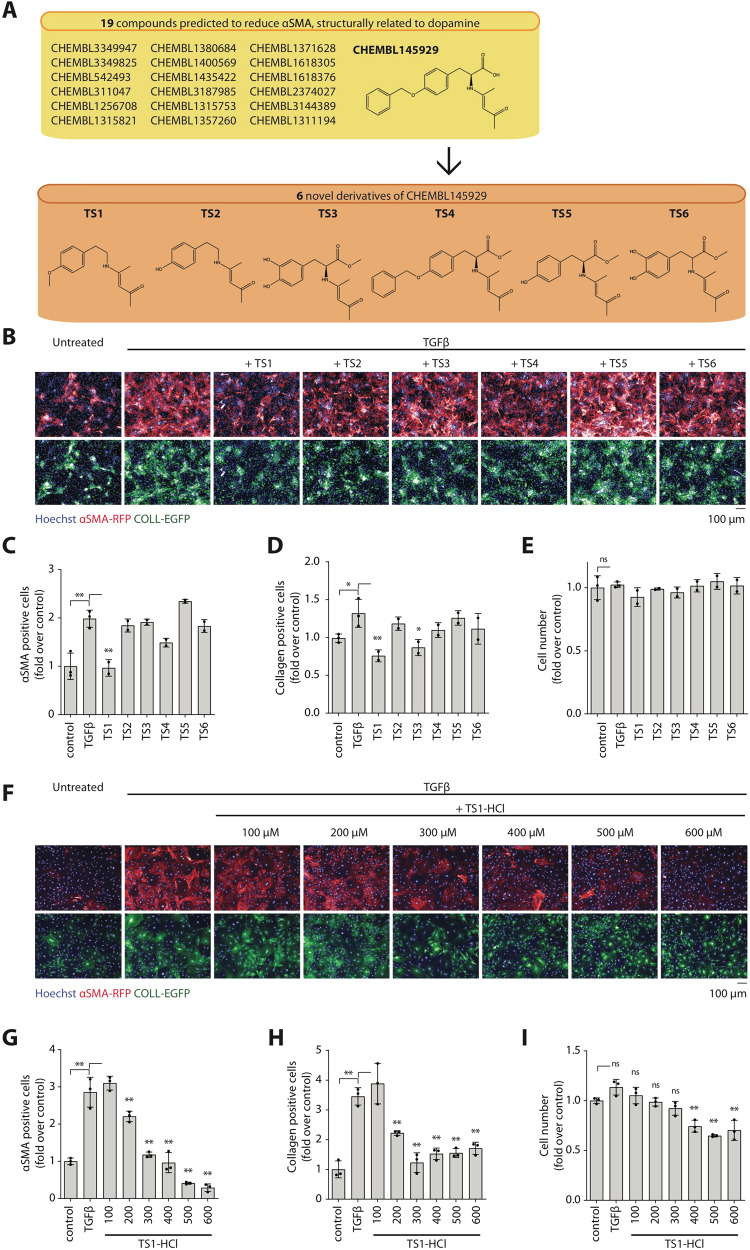
TS1 reduces activation and collagen expression of murine lung fibroblasts. **A** Flowchart showing the 19 compounds structurally related to dopamine which were predicted by machine learning to reduce αSMA expression (yellow). CHEMBL145929 was selected as the representative molecule from which six novel derivatives were synthesized, TS1-TS6 (orange). **B** Murine lung fibroblasts from αSMA-RFP, COLL-EGFP mice were treated in vitro with TGFβ and lipophilic TS1-6. Representative images are shown (Hoechst in blue, COLL-EGFP in green, αSMA-RFP in red). **C**–**E** Quantification of the number of αSMA positive cells, collagen positive cells, and total cell number (shown as fold over control). **F** Murine lung fibroblasts from COLL-EGFP/αSMA-RFP mice were treated ex vivo with TGFβ and the hydrophilic TS1-HCl. Representative images are shown (Hoechst in blue, COLL-EGFP in green, αSMA-RFP in red). **G**–**I** Quantification of the number of αSMA positive cells, collagen positive cells, and total cell number (shown as fold over control). All data are shown as mean ± SD. Statistical significance was determined using a one-way ANOVA followed by Dunnett’s multiple comparison test, *P < 0.05, **P < 0.01, *n* = 3.

To overcome the limitations of dopamine as a potential therapeutic, we aimed at synthesizing related compounds, which could be equally effective in reducing myofibroblast differentiation, but more stable and capable of reducing fibrosis in vivo. We considered the 19 dopamine-related compounds identified by our machine learning approach and chose the least sterically active and therefore expected to be the most stable one (CHEMBL145929). Based on its structure, we designed six novel derivatives, named TS1-6, shown in Fig. 3A. While dopamine is a primary amine and is thus easily oxidized, the novel derivatives are all secondary amines. This renders them more stable and suitable for in vivo administration.

We comparatively evaluated the efficacy of TS1-6 to reduce the expression of both αSMA and collagen in primary lung fibroblasts stimulated with TGFβ. As shown by the images in Fig. 3B and relative quantifications in Fig. 3c–e, TS1 was the most effective on both parameters and not toxic at 100 µM. We then produced TS1 as a hydrochloride salt, which can be dissolved in water and can therefore be easily administered in vivo. To confirm efficacy and define the optimal dose for TS1-HCl in vivo administration, we tested multiple doses in the 100−600 µM range. The 300 µM concentration significantly reduced both αSMA and collagen protein expression without overt toxicity (Fig. 3f–i) and was therefore chosen for subsequent experiments.

### TS1 is a substrate of both murine and human Drd3

**Fig. 4:**
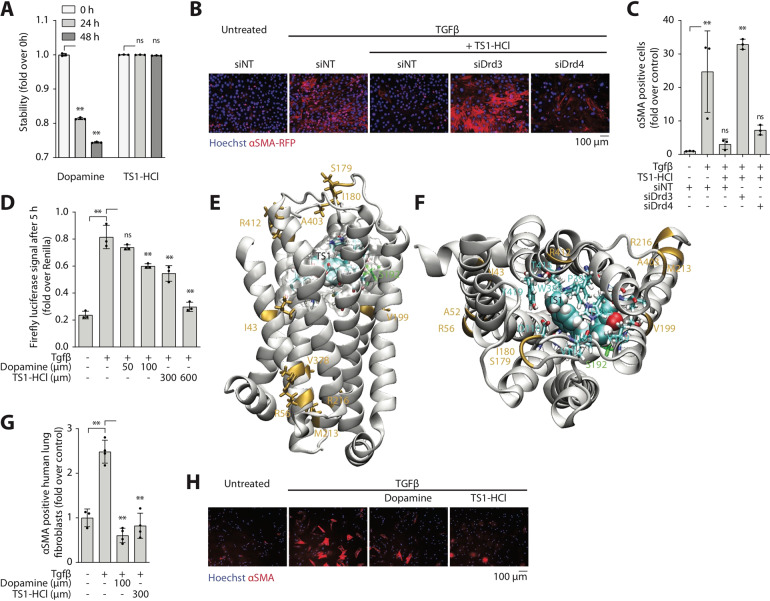
TS1 acts on the canonical TGFβ pathway via Drd3, inhibiting differentiation of both murine and human lung fibroblasts. **A** HPLC analysis shows compound stability of solubilised dopamine and TS1-HCl at room temperature over 48 h, data normalized over the initial time point (T0). **B** Murine lung fibroblasts were treated ex vivo with non-targeting siRNA (siNT) or pools of two siRNAs targeting either Drd3 or Drd4. After 48 h, cells were treated with TGFβ and TS1-HCl for an additional 48 h prior to fixation. Representative images are shown (Hoechst in blue, αSMA-RFP in red). **C** Quantification of the number of αSMA positive cells (shown as fold over control). **D** HEK293T cells stably expressing the TGFβ-sensitive (CAGA)12-luciferase reporter were treated with TGFβ and Dopamine or TS1-HCl to evaluate the effect of the compounds on the activation of the canonical TGFβ-Smad pathway. Data is the Firefly-luciferase intensity normalized over Renilla-luciferase intensity. Computational model of TS1 docked to murine Drd3: side (**E**) and top (**F**) view. Colour code: missense mutation with respect to the human receptor (yellow), residues interacting with TS1 with hydrogen bonds (green), and other interacting residues: carbons (cyan), nitrogens (blue), oxygens (red), hydrogens (white). TS1 atoms are represented with their van der Waal spheres (space-filling). **G** Human adult primary lung fibroblasts were treated ex vivo with TGFβ and TS1-HCl for 48 h prior to fixation and staining of αSMA. Quantification of the number of αSMA positive cells (shown as fold over control). **H** Representative images are shown (Hoechst in blue, αSMA in red). All data are shown as mean ± SD. Statistical significance was determined using a one-way ANOVA followed by Dunnett’s multiple comparison test, *P < 0.05, **P < 0.01, *n* = 3.

We confirmed the increased stability of TS1 compared to dopamine by HPLC analysis. While dopamine was rapidly degraded within the first 48 h, TS1 was totally stable in solution (Fig. 4A) and remained intact even after 30 days (not shown).

To confirm whether TS1 acts through the same receptor as dopamine, we silenced the two dopamine receptors expressed by lung fibroblasts (Drd3 and Drd4). While both non-targeting and anti-Drd4 siRNAs did not affect the ability of TS1 to block myofibroblast differentiation in the presence of TGFβ, the down-regulation of Drd3 completely abolished TS1 activity (Fig. 4B, C). Similar to dopamine, TS1 also acts on the canonical TGFβ pathway, as detected by a luciferase reporter of Smad 2/3 activation (Fig. 4D).

To further validate TS1 as a substrate of Drd3 through docking experiments, we first built a 3D model for the mouse receptor. The murine extracellular domain, made up of 272 residues, has never been crystallized. However, the structure of the human receptor is known [24] and differs from the murine protein by only 11 amino acids. Thus, we could model the latter with reasonable accuracy by homology (Fig. 4E, F).

TS1 was docked to the murine receptor (Supplementary Fig. 3) with a docking box covering the whole space enclosed by its helices (Supplementary Fig. 4A, B). Human Drd3 was originally crystallized with its substrate eticlopride, which was used to benchmark the method (Supplementary Fig. 4C, D). Interestingly, TS1 occupies the same binding site as eticlopride, and appears tightly wrapped by 12 residues (highlighted in Fig. 4E, F). These residues form 11 hydrophobic contacts, due to their aromatic side chains, and a hydrogen bond (2.8 Å) between Ser192 and the TS1 nitrogen (Fig. 4E, F). The position assumed by TS1 in the Drd3 channel shows that the molecule does not interact with any of the residues which differ from the human receptor (in yellow in Fig. 4E, F), suggesting that our compound could be active also in human cells.

Further docking experiments confirmed TS1 as a substrate of the human Drd3. Specifically, we identified two conformations of particular interest, suggesting that TS1 could bind more strongly to the human Drd3 than to the murine isoform. The conformation which was sampled most frequently overlapped with the one identified for the murine model (RMSD 0.3 Å). The second conformation showed more favourable interaction energies but was less populated (148 vs 892 conformers out of 2000) (Supplementary Fig. 5). While the static model predicts a strong binding of TS1 to both the murine and human receptor, it is difficult to predict the efficiency of entry into the receptor. Specifically, there is a variation in four amino acids between the murine and human receptor in a flexible loop that could influence the accessibility of the binding site.

Supported by docking results, we tested the activity of TS1 on human adult lung fibroblasts and confirmed that it significantly inhibits TGFβ-induced myofibroblast activation in vitro, similar to dopamine (Fig. 4G, H).

### TS1 reduces myofibroblast activation and fibrosis in vivo

**Fig. 5:**
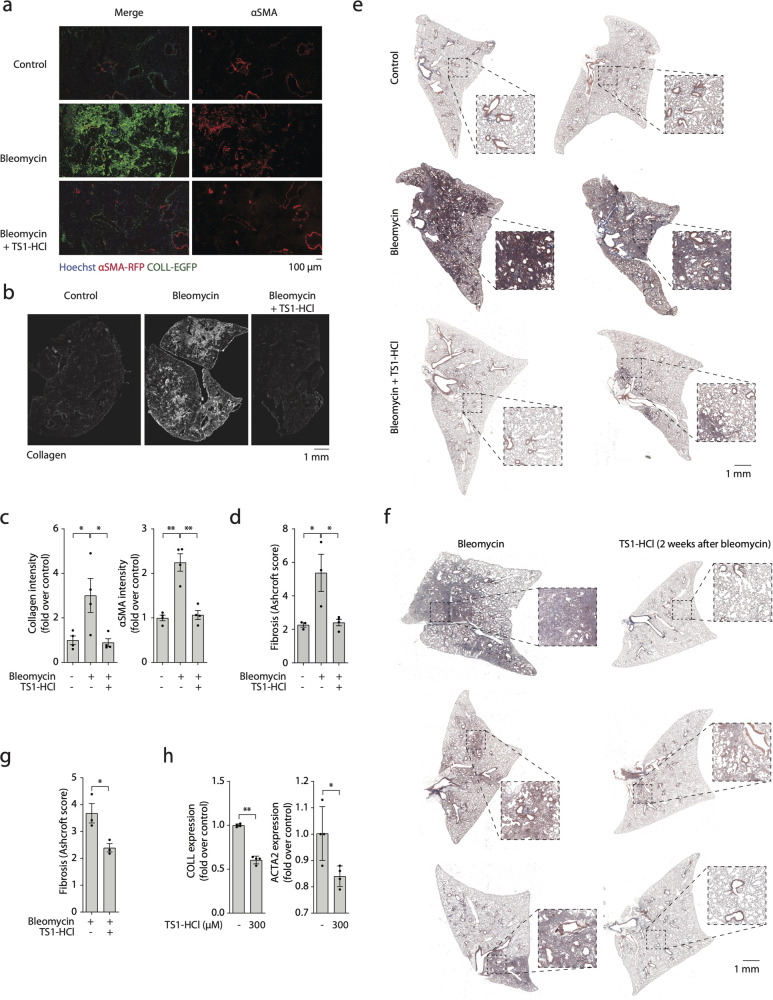
TS1 inhibits myofibroblast differentiation in vivo and reduces lung fibrosis in bleomycin-treated mice. **a** COLL-EGFP/αSMA-RFP mice were treated with bleomycin and two days later with TS1-HCl by intratracheal injection; lungs were harvested after 14 days. Representative images are shown, with nuclei shown in blue, COLL-EGFP in green, and αSMA-RFP in red. **b** Representative whole organ images of treated mice, in which collagen expression is shown in white. **c** Quantification of collagen and αSMA intensity of lungs shown in panels (**a**, **b**), normalized over untreated, control lungs. **d**, **e** Mice were treated with bleomycin, either alone or in combination with TS1-HCl by intratracheal injection. Lungs were harvested after 30 days and stained with Masson-Trichrome to visualise collagen fibres in blue. Lung fibrosis was quantified using the Ashcroft score. **f** Mice were treated with bleomycin, and after 2 weeks mice were treated with TS1-HCl by intratracheal injection. Lungs were harvested after a total of 30 days and stained with Masson-Trichrome to visualise collagen fibres in blue. **g** Quantification of lung fibrosis was performed using the Ashcroft score to quantify collagen fibres stained in blue. **h** Levels of expression of collagen (*col1a1*) and αSMA (*acta2*) in human fibroblasts derived from IPF lungs, upon treatment with TS1-HCl at the indicated dose. Results are normalized to *Gapdh* expression. All data are shown as mean ± S.E.M. Statistical significance was determined using a one-way ANOVA followed by Dunnett’s multiple comparison test, *P < 0.05, **P < 0.01.

Finally, we tested the capacity of TS1 to effectively suppress the formation of lung fibrosis in vivo. To visualize the effect of TS1 on myofibroblast differentiation, we injected TS1 intra-tracheally two days after the administration of bleomycin in COLL-EGFP αSMA-RFP mice. As expected, bleomycin strongly increased the expression of both collagen and αSMA by lung fibroblasts. Both genes were markedly reduced in mice treated with TS1 (Fig. 5a). While the effect on αSMA was comparable to that of dopamine, TS1 was by far more potent in reducing the expression of collagen, as shown by the details in Fig. 5a and the whole organ section in Fig. 5b. Quantification of both collagen and αSMA protein expression both in the presence and in the absence of TS1 is reported in Fig. 5c. Reduced myofibroblast differentiation by TS1 resulted in a significant decrease in lung fibrosis at 30 days, as quantified in Fig. 5d and shown by whole organ sections in Fig. 5e. Finally, we also assessed the value of TS1 as a therapeutic drug, by administering it in lungs with established fibrosis. For this purpose, we treated mice with bleomycin, and then we administered TS1 two weeks following bleomycin delivery. This treatment significantly reduced the amount of fibrosis at 21 days (Supplementary Fig. 6) and at 30 days (Fig. 5f, g) as quantified using the Ashcroft score. Intriguingly, the levels of bleomycin-induced inflammation and fibrosis were more pronounced at 21 days than at 30 days, allowing TS1 to induce a more significant reduction at this earlier time point. Moreover, as the Ashcroft score reveals, TS1 induces a continued improvement in the reduction of fibrosis between 21 and 30 days.

To further explore the therapeutic potential of TS1 we assessed its capacity to inhibit the activation of myofibroblasts harvested from the lungs of IPF patients. As shown in Supplementary Fig. 7, TS1 reduced the expression of both collagen and αSMA upon cell stimulation with TGFβ. More impressive and significant results were observed in the absence of exposure to TGFβ (Fig. 5h), consistent with chronic activation of myofibroblasts in IPF lungs.

Thus, TS1 reverses myofibroblast differentiation and fibrosis, both in preventive and curative approaches in mice, as well as in human IPF lung fibroblasts.

## Discussion

Our work describes a unique wet-dry-wet approach for drug discovery using myofibroblast differentiation as a phenotypic readout. We started from a “wet” drug screen using a library of 640 compounds, an extensive library that nevertheless fails to represent the full scope of potentially therapeutic compounds. We then applied a “dry” machine learning strategy to extrapolate our wet results onto a database of over 1 million drug-like compounds to eventually design and synthetize a few novel candidates, one of which (TS1) potently inhibited lung fibrosis in a “wet” context. The power and efficiency of our approach is documented by the relatively low number of candidates needed to be produced and validated in vitro prior to moving to in vivo models.

Both academic institutions and pharma industry are investing high resources in the design and execution of virtual compound screens [25–27]. The ability to process large datasets using machine learning can be successfully applied in the prediction of druggable targets and is often combined with computational biology approaches that take the global properties of a compound into consideration [28]. High throughput data were used as a powerful data source for machine learning analysis in multiple contexts; for example, to improve the analysis of protein localization within cells and to search for novel antimicrobial compounds [29–31]. Our work provides further evidence that virtual screening can be a powerful tool to identify therapeutic compounds quickly and effectively.

Our virtual screening pointed to the selection of a number of molecules that were structurally related to dopamine, yet dopamine itself proved to be inefficient at reducing fibrosis in vivo. This can be explained through the low stability of dopamine in vivo, both due to the instable chemical structure and the presence of degrading enzymes in circulation. TS1 is a much more stable compound, which has demonstrated success in vitro and in vivo in reducing both αSMA and collagen expression levels, as well as counteracting the development of fibrosis both in a preventative and therapeutic protocol. Our docking and siRNA-mediated knock-down experiments have shown that this derivative acts through the Drd3 receptor, and we have further demonstrated a direct effect on inhibiting the pro-fibrotic canonical TGFβ pathway.

In the original “wet” screen, we identified haloperidol as a potent anti-fibrotic agent [11]. Interestingly, haloperidol is known to be a dopamine receptor antagonist, which could be seen as a contradiction to our findings. However, we have previously demonstrated that haloperidol inhibits myofibroblast differentiation by acting on sigma receptor 1, and moreover this antipsychotic drug has been shown to specifically inhibit Drd2, but not Drd3 or Drd4 [32]. In our work, we have shown Drd2 to be expressed below detectable levels in both lung and heart fibroblasts and therefore believe that TS1 is working through a different pathway than haloperidol.

While there are contradictory opinions on the role of dopamine agonists in fibrosis, there is strong evidence to support an anti-fibrotic effect of Drd3. One publication shows the anti-fibrotic effect of the Drd3 agonist pramipexole in a model of cardiac fibrosis induced by morphine, which activates Smad 2/3 signalling [33]. Additionally, more definitive evidence comes from Drd3 knock-out mice. Removal of this receptor in vivo results in increased cardiac fibrosis, mimicking the phenotype of aged animals [34].

Our results are complemented by a recent publication, showing that dopamine and various dopamine receptor agonists have an anti-fibrotic effect, by acting through the Drd1 receptor to inhibit the pro-fibrotic Yap/Taz pathway [35]. In this work, the main compound which is investigated is dihydrexidine, a moderately selective agonist able to bind both D1- and D2-type dopamine receptors. We have shown barely detectable expression levels of Drd1 in lung fibroblasts. This would possibly suggest a combined mechanism by which both Drd1 and Drd3 could contribute to the anti-fibrotic effect of dopamine receptor agonists, whereby the first is acting via the Yap/Taz pathway while the latter has an effect on canonical TGFβ signalling. A further interesting observation made by Haak et al. is a dramatic increase in the expression of Drd3 in lung epithelial cells following bleomycin treatment. This could further implicate Drd3 as an important pathway in fibrosis, indicating a compensatory role in mitigating the fibrotic response.

In conclusion, our work demonstrates the power of transdisciplinary approaches in the identification of novel anti-fibrotic drugs. By combining HTS and high content analysis, with machine learning and chemical synthesis, we were able to develop new powerful anti-fibrotic tools. Besides repurposing the known FDA-approved compound dopamine, we also identified and produced a novel derivative, TS1. Our data show that TS1 is highly stable and shows promising results both in vitro and in vivo in fighting fibrosis through direct modulation of the canonical TGFβ signalling pathway.

## Materials and methods

### Machine learning

Starting from previously published data [11], we built a predictive model that takes compound descriptions as input and consequently predicts their effect on αSMA and collagen expression. The original screen was used as the training set. It included 640 compounds, identified with CAS numbers. In order to match CAS numbers with other identifiers, the Chemical Translation Service (http://cts.fiehnlab.ucdavis.edu/) and some manual checking were used. Each molecule was eventually described using Extended Connectivity Fingerprints (ECFP)—represented by a binary vector indicating whether a given compound contains a set of substructures. Bagging ensembles of predictive clustering trees (PCTs) were used as predictive models, as they yield state-of-the-art predictive performance and can provide estimates of the reliability of their predictions. We used 10-fold cross-validation to estimate the predictive performance of the models on unseen compounds. Since the goal was to identify interesting candidates for wet-lab experiments, we also wanted to investigate the reliability of individual predictions. For this, we looked at the standard deviation of predictions of individual trees in the ensemble and found a clear correlation between lower standard deviation of the predictions of individual trees and lower prediction error of the ensemble prediction. We identified ChEMBL (version 24) as a suitable database of compounds to use as a testing set and apply the predictive model on, in search of interesting candidates.

### Cell culture and treatments

Primary murine fibroblasts were isolated from lung and heart of αSMA-RFP C57BL/6 or αSMA-RFP/COLL-EGFP C57BL/6 mice by tissue digestion and mechanical shearing. For this, tissue was minced and then digested for 1 h at 37 °C with a magnetic stirrer using collagenase, dipeptidase, and accutase. Dissociated tissue was passed through a 70 µm cell strainer and centrifuged at 450 × *g*. The resulting cell pellet was resuspended in DMEM with 10% FBS, and fibroblasts were allowed to attach on plastic tissue culture plates for 2 h. Medium was changed, and cells were left to recover overnight in a humidified incubator at 37 °C before being immediately used for experiments. Human primary lung fibroblasts were purchased from ATCC, while those from IPF lungs were obtained from Dr. Steven Huang (University of Michigan). Human recombinant TGFβ1 (Peprotech) was used for stimulation in combination with drug treatments, and cells were fixed after 48 h. Zanamivir, topiramate and dopamine were purchased from Sigma and resuspended in water or DMSO, as required. Human cells were stained with αSMA-Cy3 (Sigma-Aldrich, clone 1A4).

### Transfections

Silencing of Drd3 and Drd4 was performed in primary murine fibroblasts using pools of two siRNAs purchased from Sigma (siDrd3: SASI_Mm01_00104662, SASI_Mm01_00104663; siDrd4: SASI_Mm01_00044753, SASI_Mm01_00044754). Control cells were transfected with ON-TARGETplus Non-targeting Control siRNA #4 (Dharmacon). Cells were transfected using 50 nM of siRNA complexed with lipofectamine RNAiMAX (Thermofisher).

### Immunohistochemistry

Cells were fixed with 4% PFA and nuclei were stained using Hoechst 3334. Images were acquired using an Operetta high content screening microscope (Perkin Elmer) with a 20× objective. A minimum of 9 fields were acquired per well, and the number of αSMA positive cells was quantified using the Columbus software (threshold set for TRITC channel cellular mean fluorescence intensity). For imaging of tissue sections from αSMA-RFP/COLL-EGFP C57BL/6 mice, immunohistochemistry was performed on cryosections fixed with 4% PFA or paraffin embedded sections fixed with formalin, which were deparaffinised before processing. Nuclei were counterstained with Hoechst and tissue was imaged on a Nikon Eclipse Ti-E inverted fluorescence microscope. For visualization of fibrosis, tissue was stained using Masson-Trichrome (Bio Optica).

### qRT-PCR

RNA was prepared using Trizol (Invitrogen) and phenol chloroform extraction, and cDNA was prepared using the First strand cDNA synthesis kit (Thermo Scientific) following the manufacturer’s instructions. Samples were run on a C1000 Thermal Cycler (Biorad) and analyzed using the proprietary software and the 2^-dCT and 2^-ddCT methods.

Primers for dopamine receptors were taken from literature [36]: *mDrd1* fw CACGGCATCCATCCTTAACCT, rev TGCCTTCGGAGTCATCTTCCT; *mDrd2* fw ACCTGTCCTGGTACGATGATG, rev GCATGGCATAGTAGTTGTAGTGG; *mDrd3* fw CCAGTTCACTATCAGCATGGC, rev CCCCTGTTGTGTTGAAACCAA; *mDrd4* fw GGTGTCGGACCCTACTCAG, rev GGCAGGACTCTCATTGCCTT; *mDrd5* fw TGCTGTCCAATGAGACACCC, rev GATGGCGTAGGTTCGGTTCAG. Human primers were the following: *col1a1* fw AAGGGACACAGAGGTTTCAGTGG, rev CAGCACCAGTAGCACCATCATTTC; *acta2* fw GTGAAGAAGAGGACAGCACTG, rev CCCATTCCCACCATCACC. *Gapdh* was used as a housekeeping gene for both human and mouse experiments.

### Luciferase reporter assay

HEK293T cells stably expressing the TGFβ-sensitive (CAGA)12-luciferase reporter (pGL3 CAGA12 LC + PuroR-P2A-Renilla-T2A-EGFP) [37] were starved overnight in 0% FBS in low glucose DMEM, and then treated for 6 h with human recombinant TGFβ1 (Peprotech), in combination with various concentrations of dopamine or TS1-HCl. Cells were lysed with Glo-lysis buffer (Promega) and assayed using the Dual-Glo luciferase kit (Promega) on the Wallac Envision plate reader (Perkin Elmer).

### Animal experiments

Ethical and experimental procedures were reviewed and approved by the ICGEB Animal Welfare board, meeting the requirements of the EU Directive 2010/63/EU, and by the Italian Ministry of Health (approval number 806/2018-PR). Bleomycin was injected intratracheally at 1 U/kg, dopamine was administered at 0.76 µg/kg, TS1-HCl at 2 mg/kg. Mice were sacrificed at either 14 or 30 days post-treatment. The severity of fibrosis was determined on Masson-Trichrome stained lung sections using the Ashcroft score [38].

### Compound synthesis

Commercially available chemicals were of reagent grade and used as received. Reaction courses and product mixtures were routinely monitored by thin-layer chromatography (TLC) on silica gel pre-coated F_254_ Merck plates. Melting points were determined using a Stuart SMP300 apparatus and uncorrected. Infrared spectra were recorded on a Jasco 4700 spectrophotometer in nujol mulls. Nuclear magnetic resonance spectra were registered on a Varian 400 MHz (400 for ^1^H-NMR and 101 MHz for ^13^C-NMR), shown in Supplementary Figure 8. Chemical shifts are reported as δ (ppm) in CDCl_3_ solution (δ = 7.26 ppm for ^1^H-NMR and δ = 77.2 for ^13^C-NMR) or DMSO-d6 (δ = 2.49 ppm for ^1^H-NMR and δ = 39.52 for ^13^C-NMR); 20 µL of D_2_O was added to assign NH and OH protons. Microanalyses (C, H, N) were carried out using an Elementar Vario ELIII apparatus and were in agreement with theoretical values ± 0.4%. ESI-MS spectra (LRMS) were recorded on a Bruker Daltonics Esquire 4000 spectrometer by infusion of a solution of the sample in MeOH (HPLC grade). Microwave oven synthesizer Anton Paar monowave 400.

### Compound stability analysis

HPLC analysis was performed using methanol and ammonium acetate (Sigma Aldrich). Analyses were performed on an HPLC Agilent 1260 Infinity II with a Supelco C18 Discovery 250 × 4.6 mm, 5 mm particle size (Supelco) column with a column guard and an autosampler using a DAD detector at 210 nm. The flow was set to 1.00 mL/min with an injection volume of 20 µL. The mobile phase was ammonium acetate 0.01 M and methanol (60:40) using isocratic condition at 25 °C.

### Homology modelling and docking

Drd3 murine (UniProt P30728) and human (UniProt P35462), were aligned with BLAST [39]. Three-dimensional receptor models were built by homology model with SWISS-MODEL [40] via the ExPASy web server by employing PDB ID 3PBL [24] as a template (human dopamine D3 receptor, Lysozyme chimera, in complex with eticlopride). TS1 SMILES was converted into a protonated three-dimensional model with Obabel [41], and minimized with AM1 as implemented in MOPAC [42]. The Lamarckian Genetic Algorithm (LGA) was used for docking TS1 to both human and murine Drd3 models. The docking box (50 × 50 × 50), spacing 0.375 Å, was centred at (2.692, −16.966, 0.876) in the frame of the original template (PDB ID 3PBL) coordinates [24]. 2000 LGA runs with initial population size of 500, 10,000,000 maximum numbers of evaluations, and standard parameters were run as implemented in AutoDock [43]. Docked conformations were clustered with 2.0 Å tolerance. For both murine and human models, the representative conformations of the larger cluster (comprising ≈45% of the generated conformations) were selected as representative conformation for subsequent analysis. Specific ligand−protein interactions were derived through LigPlot+ [44].

## Supplementary information

Supplemental Figures and Methods

checklist

Confirmation from all authors

## Data Availability

The data supporting the findings from this study are available within the manuscript and its supplementary information. Source data are provided with this paper.
