# Supplementary material for: Wet-dry-wet drug screen leads to the synthesis of TS1, a novel compound reversing lung fibrosis through inhibition of myofibroblast differentiation
Source: Cell Death Dis. 2021 Dec 17;13(1):2. doi: 10.1038/s41419-021-04439-4 (PMC8677786; doi:10.1038/s41419-021-04439-4)
Supplement: Supplementary file 1 — Supplemental Figures and Methods [file 41419_2021_4439_MOESM1_ESM.pdf]

Wet-dry-wet drug screen leads to the synthesis of TS1, a novel compound inhibiting lung fibrosis

Supplementary material

Supplementary Figures

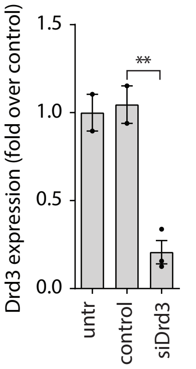

Supplementary Figure 1. Knockdown of Drd3 using siRNA significantly reduces receptor levels in murine lung fibroblasts. RT-PCR analysis shows the reduction of Drd3 in murine lung fibroblasts, following treatment with a pool of two siRNAs targeting Drd3. A non-targeting siRNA was used as control. Results are normalised to Gapdh expression. Data are shown as mean ± SD. Statistical significance was determined using a one-way ANOVA followed by Dunnett's multiple comparison test, **P < 0.01, n = 3.

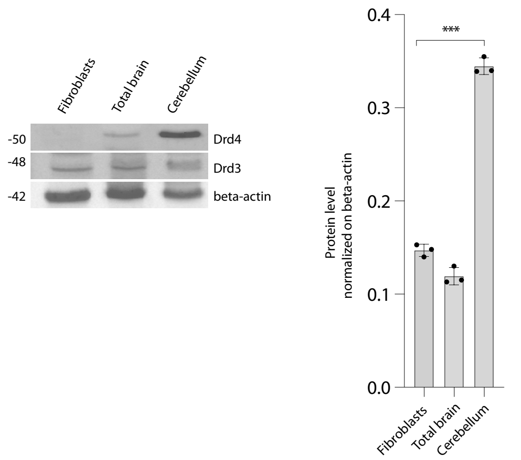

Supplementary Figure 2. Drd3 but not Drd4 protein is expressed by fibroblasts. Western blot analysis was performed to analyse the protein expression of Drd3 and Drd4 in murine lung fibroblast, cerebellum and whole brain lysate. Quantifications are normalised on beta-actin protein expression levels. Data are shown as mean ± SD. Statistical significance was determined using a student's t-test, ***P < 0.01, n = 3.

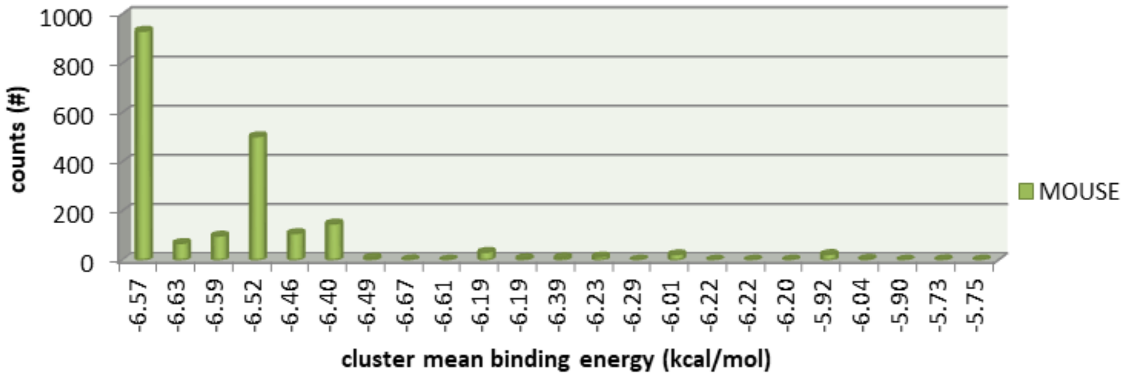

Supplementary Figure 3. Docking Results for the murine Drd3 model: cluster distribution of poses as a function of their cluster average estimated energy.

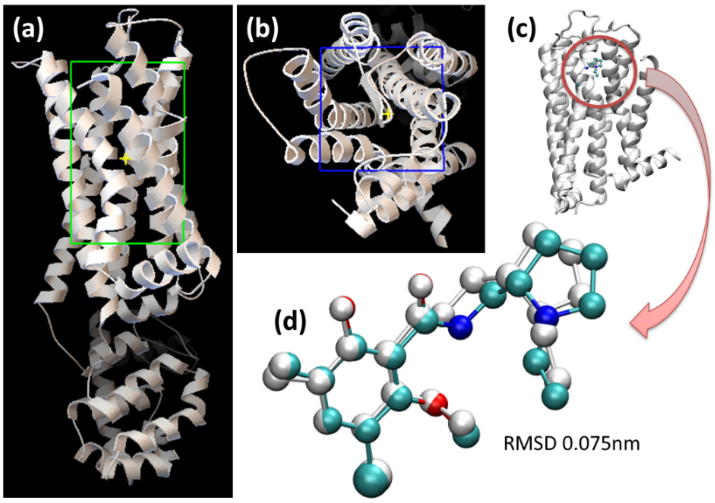

Supplementary Figure 4. Docking setup and method validation: (a,b) side and top view of the selected bocking box, (c) eticlopride and homology DRD3 human model docking result and (d) comparison between eticlopride in 3PBL crystal structure (white) and docking result (color).

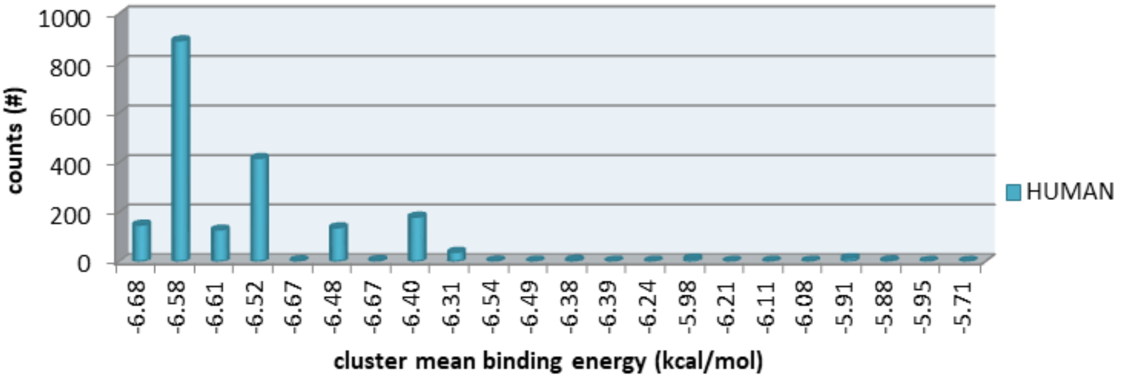

Supplementary Figure 5. Docking results for the human DRD3 model: cluster distribution of poses as a function of their cluster average estimated energy.

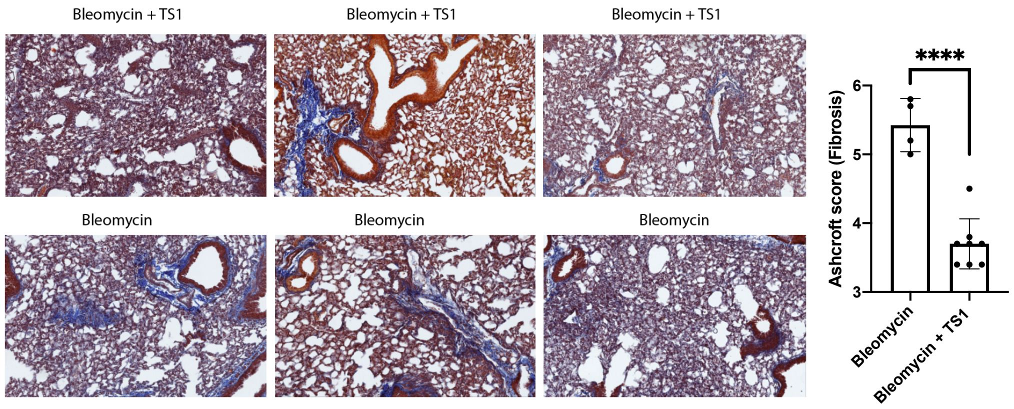

Supplementary Figure 6. TS1 administered in a therapeutic protocol shows anti-fibrotic effects already after 1 week. Mice were treated with bleomycin, and TS1 was administered two weeks later. Lungs were harvested at 21 days and fibrosis was quantified using the Ashcroft score. TS1 treatment significantly reduced the amount of fibrosis. Data are shown as mean ± SEM. Statistical significance was determined using a student's t-test, ****P < 0.0001, n = 3.

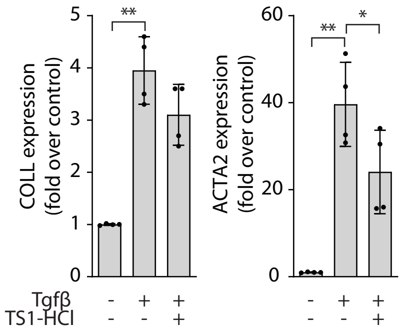

Supplementary Figure 7. TS1 reduced the expression of both collagen and αSMA in human fibroblasts from idiopathic pulmonary fibrosis patients, after stimulation with TGFβ. Human fibroblasts from idiopathic pulmonary fibrosis patients were stimulated with TGFβ and treated with TS1. Using qRT-PCR analysis, a mild reduction of collagen expression and a significant reduction of ACTA2 expression were observed.

(*E*)-4-((4-methoxyphenetyl)amino)pent-3-en-2-one (TS-1)

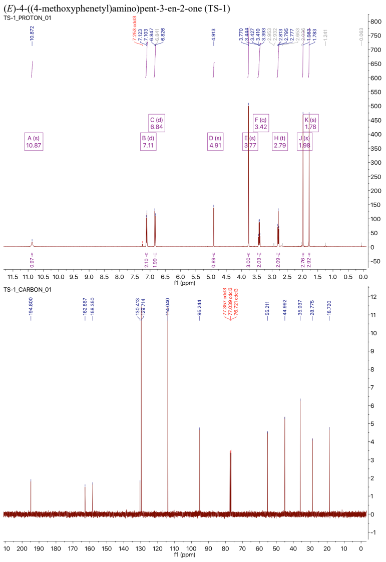

(*E*)-4-((4-hydroxyphenetyl)amino)pent-3-en-2-one (TS-2)

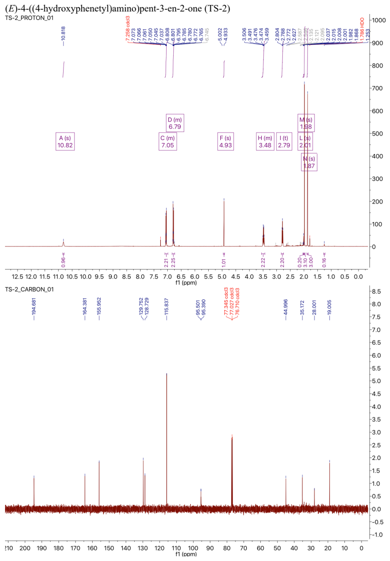

(S,*E*)-3-(3,4-dihydroxyphenyl)-2-((4-oxopent-2-en-2-yl)amino)propanoate (TS-3)

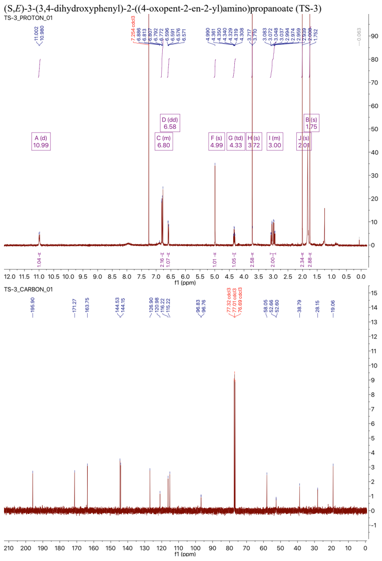

Methyl (S,*E*)-3-(4-(benzyloxy)phenyl)-2-((4-oxopent-2-en-2-yl)amino)propanoate (TS-4)

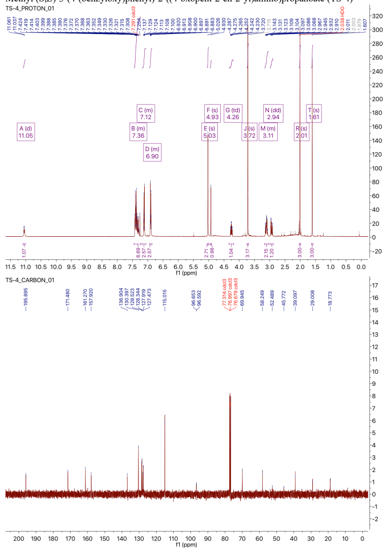

Methyl (S,*E*)-3-(4-hydroxyphenyl)-2-((4-oxopent-2-en-2-yl)amino)propanoate (TS-5)

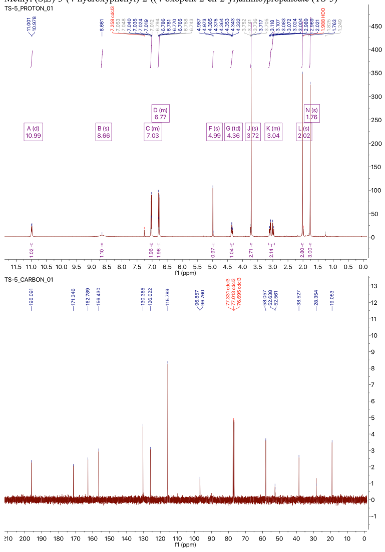

Methyl (*E*)-3-(3,4-dihydroxyphenyl)-2-((4-oxopent-2-en-2-yl)amino) propanoate (TS-6)

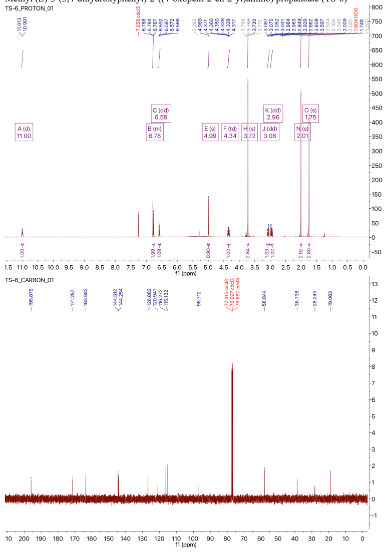

Supplementary Figure 8. NMR spectra for TS1-TS6

Supplementary Methods

Chemical compound synthesis

Coupling constants (*J*) are reported in Hz and the splitting abbreviations used are: s, singlet; d, doublet; dd, doublet of doublets; t, triplet; td, triplet of doublets; q, quartet; qd, quartet of doublets; m, multiplet; br, broad

Additional abbreviations used are:
CDCl 3 : deuterated chloroform
D 2 O: deuterated water
DCM: dichloromethane
DMF: dimethylformamide
ES-MS: electron-spray mass spectrometry
Et 3 N: triethyl amine
EtOH: ethanol
IR: infrared spectroscopy
MeOH: methanol
Mp: melting point
MS: mass spectrometry
MW: molecular weight
NMR: nuclear magnetic resonance
Rf: retention factor
TLC: thin layer chromatography

Synthetic procedure for TS1 and TS2 derivatives
With the use of 4-methoxyphenetylamine, L-tyrosine, acetyl acetone and triethylamine, **TS1** and **TS2** were obtained.
TS1 was synthesized using the corresponding 4-methoxyphenetylamine and acetyl acetone under reflux condition. **TS2** was prepared starting from L-tyrosine as amine but using a microwave oven synthesizer and the reaction included a decarboxylation process.

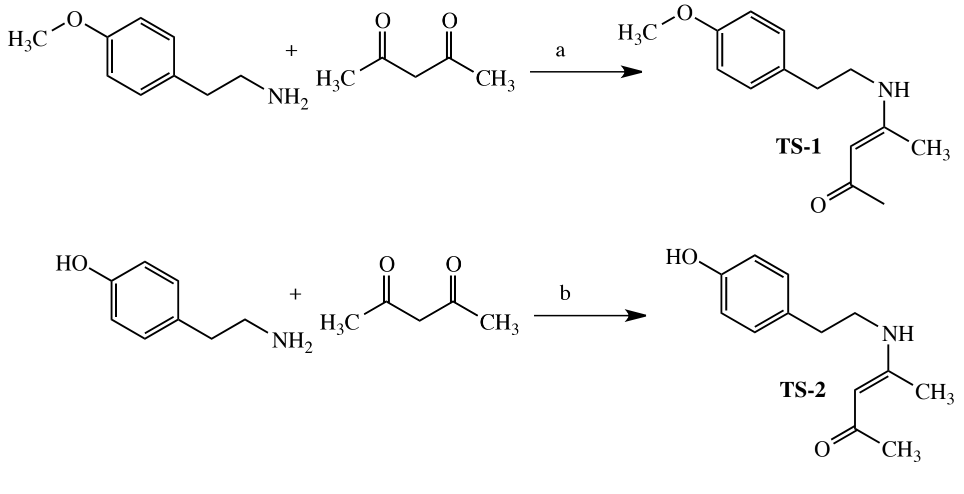

Reagents and conditions: a) EtOH, reflux, 17h; b) DMF, MW 3 cycles

- **Synthesis of TS1: (E)-4-((4-methoxyphenetyl)amino)pent-3-en-2-one**

A solution of 100 mg (0.66 mmol) of 4-methoxyphenetylamine in 10 mL of $EtOH_{abs}$ was treated with 66.2 mg (0.66 mmol) of acetylacetone and the resulting mixture was allowed to stir at reflux temperature for 17 hours, monitored by TLC (DCM – EtOH 9.8:0.2). Once the reaction was completed, the solvent was removed under reduced pressure to afford a chromatographically pure yellow oil, of **TS1**.

MW: 233.31 (g/mol); **Yield**: 97%; **Mp**: n.d.; **Rf**: 0,45 (DCM - EtOH 9.5 : 0.5); **IR**: (nujol, cm^{-1}): 3419 (NH), 1612 (C=O). **^{1}H-NMR** ($CDCl_3$, δ, ppm): 10.87 (s, 1H, NH), 7.11 (d, *J* = 8.5 Hz, 2H, arom.), 6.84 (d, *J* = 8.6 Hz, 2H, arom.), 4.91 (s, 1H, CH), 3.77 (s, 3H, OCH_3), 3.42 (q, *J* = 7.1 Hz, 2H, CH_2), 2.79 (t, *J* = 7.2 Hz, 2H, CH_2), 1.98 (s, 3H, CH_3), 1.78 (s, 3H, CH_3); **^{13}C-NMR** ($CDCl_3$, δ, ppm): 194.92, 162.99, 158.47, 130.53, 129.84, 114.16, 95.37, 55.33, 45.11, 36.06, 28.89, 18.84. **MS**: *m/z* 234 $[M+H]^+$, 256 $[M+Na]^+$. Anal. calcd for $C_{14}H_{19}NO_2$ (%): C, 72.07; H, 8.21; N, 6.00. Found: C, 72.20; H, 8.34; N, 5.85. This compound was dissolved in a minimum amount of anhydrous Et_2O, at 0°C, and treated with gaseous HCl to obtained the corresponding hydrochloride as a light-yellow solid (**Mp**: 190°C dec).

- **Synthesis of TS2: (*E*)-4-((4-hydroxyphenetyl)amino)pent-3-en-2-one**

The compound has been synthesized using a microwave oven reactor, with the following procedure: In a 4 mL sealed-vessel, 50.0 mg (0.27 mmol) of L-tyrosine were dissolved in 1 ml of DMF and 27.6 mg (1 eq) of acetyl acetone were added. The mixture was subjected to three cycles of MW irradiation: 150°C/500W/18 bar/18 minutes, 200°C/600W/20/18 minutes and finally 200°C/600W/20 bar for 13 minutes. The solvent was removed under *vacuum* and the residue dissolved in 10 mL of DCM and washed with distilled water (3 x 10 mL). The collected organic layers were dried over Na_2SO_4, filtered and evaporated under reduced pressure to afford a light orange solid.

MW: 219.28 (g/mol); **Yield**: 51%; **Mp**: 109-111°C; **Rf**: 0.27 (DCM - EtOH 9.5 : 0.5); **IR**: (nujol, cm^{-1}): 3434 (NH), 1606 (C=O). **^{1}H-NMR** ($CDCl_3$, δ, ppm): 10.82 (s, 1H, NH), 8.68 (s, 1H, OH), 7.08 – 7.03 (m, 2H, arom.), 6.81 – 6.77 (m, 2H, arom.), 4.93 (s, 1H, CH), 3.53 – 3.45 (m, 2H, CH_2), 2.79 (t, J = 6.4 Hz, 2H, CH_2), 1.98 (s, 3H, CH_3), 1.87 (s, 3H, CH_3). **^{13}C-NMR** ($CDCl_3$, δ, ppm): 194.68, 164.38, 155.95, 129.75, 128.73, 115.84, 95.50, 95.39, 45.00, 35.17, 28.00, 19.01. **MS**: *m/z* 220 $[M+H]^+$. Anal. calcd for $C_{13}H_{17}NO_2$ (%): C, 71.21; H, 7.81; N, 6.39. Found: C, 72.04; H, 7.50; N, 6.15.

Synthetic procedure for TS3, TS4, TS5 and TS6 derivatives

With the use of L-Dopa, D,L-Dopa, *O*-benzyl-L-tyrosine and L-tyrosine, the corresponding **TS3, TS4, TS5** and **TS6** compounds were obtained.

The aforementioned acid reactants were treated with $SOCl_2$ in MeOH to afford the corresponding methyl ester which were subsequently made react with acetyl acetone/Et_3N in $EtOH_{abs}$ to afford the corresponding final compounds.

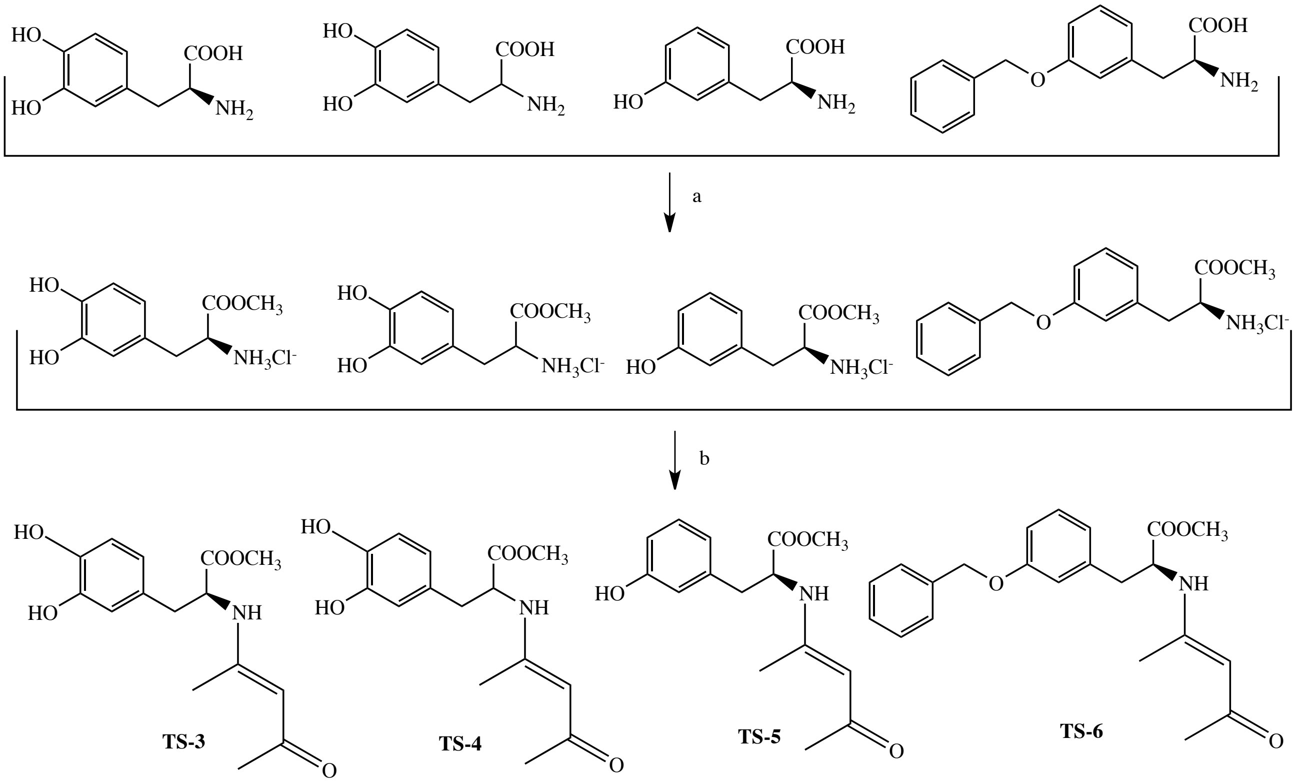

Reagents and conditions: a) MeOH/$SOCl_2$, (Ar) reflux, 6h; b) Acetyl acetone, Et_3N, EtOH, reflux, 40-58h.

- **General procedure for the synthesis of compounds TS3, TS4, TS5 and TS6, starting with the synthesis of (S)-methyl 2-amino-3-(3,4-dihydroxyphenyl)propanoate HCl**

In a double-neck round bottom flask, 1.00 g (5.11 mmol) of L-Dopa was dissolved in 25 ml of MeOH and the solution was stirred at 0°C in an ice-bath under Ar atmosphere. $SOCl_2$ (1.01 g, 8.52 mmol) was then added dropwise and the solution was allowed to reach room temperature. After few minutes the reaction was heated at reflux for 6 hours and monitored by TLC (DCM-EtOH 6:4). When the reaction was completed, the solvent was removed by reduced pressure and the residue triturated with Et_2O to afford a chromatographically pure white solid of (S)-methyl 2-amino-3-(3,4-dihydroxyphenyl)propanoate HCl.
MW: 247.68 (g/mol); **Yield**: 87%; **Mp**: 166-168 °C; **Rf**: 0.61 (DCM - EtOH 6 : 4); **IR**: (nujol, cm^1): 1750 (C=O);
^{1}H-NMR (DMSO, δ, ppm): 8.93 (s+s, 2H, 2xOH), 8.53 (s all., 3H, NH_3^+), 6.66 (d, J = 7.9 Hz, 1H, arom.), 6.57 (d, J = 2.2 Hz, 1H, arom.), 6.43 (dd, J = 8.0, 2.2 Hz, 1H, arom.), 4.09 (dd, J = 6.9, 5.9 Hz, 1H, CH), 3.67 (s, 3H, OCH_3), 3.06 – 2.82 (m, 2H, CH_2). **MS**: *m/z* 249 $[M+H]^+$.

With the same procedure the following esters were obtained:

- **Methyl 2-amino-3-(3,4-dihydroxyphenyl)propanoate HCl**

White resinous solid; **MW**: 247.68 (g/mol); **Yield**: 98%; **Mp**: n.d.; **Rf**: 0.64 (DCM - EtOH 6:4); **IR**: (nujol, cm^{-1}): 1734 (C=O); **^{1}H-NMR** (DMSO, δ, ppm): δ 8.93 (s+s, 2H, 2xOH), 8.51 (s all., 3H, NH_3^+), 6.66 (d, *J* = 7.9 Hz, 1H, arom.), 6.57 (d, *J* = 2.2 Hz, 1H, arom.), 6.43 (dd, *J* = 8.0, 2.2 Hz, 1H, arom.), 4.09 (t, *J* = 6.5 Hz, 1H, CH), 3.66 (s, 3H, OCH_3), 3.03 – 2.84 (m, 2H, CH_2). **MS**: *m/z* 249 $[M+H]^+$.

- **(S)-Methyl 2-amino-3-(4-(benzyloxy)-3-hydroxyphenyl)propanoate HCl**

White solid; **MW**: 321,80 (g/mol); **Yield**: 99%;; **Mp**: 196-8°C; **Rf**: 0.71 (DCM - EtOH 6 : 4); **IR**: (nujol, cm^{-1}): 1742 (C=O); **^{1}H-NMR** (DMSO, δ, ppm): 8.58 (s all., 3H, NH_3^+), 7.44–7.27 (m, 5H, arom.), 7.13 (d, *J* = 8.6 Hz, 2H, arom.), 6.95 (d, *J* = 8.7 Hz, 2H, arom.), 5.06 (s, 2H, CH_2), 4.19 (t, *J* = 6.5 Hz, 1H, CH), 3.65 (s, 3H, OCH_3), 3.05 (qd, *J* = 14.2, 6.5 Hz, 2H, CH_2). **MS**: *m/z* 323 $[M+H]^+$.

- **(S)-Methyl 2-amino-3-(4-hydroxyphenyl)propanoate HCl**

White solid; **MW**: 231,68 (g/mol); **Yield**: 99%; **Mp**: 191-3°C; **Rf**: 0.68 (DCM-EtOH 6:4); **IR**: (nujol, cm^{-1}): 1742 (C=O); **^{1}H-NMR** (DMSO, δ, ppm): 9.42 (s, 1H, OH), 8.51 (s all., 3H, NH_3^+), 6.98 (d, *J* = 8.5 Hz, 2H, arom.), 6.69 (d, *J* = 8.5 Hz, 2H, arom.), 4.15 (t, *J* = 6.4 Hz, 1H, CH), 3.65 (s, 3H, OCH_3), 2.99 (qd, *J* = 14.2, 6.6 Hz, 2H, CH_2). **MS**: *m/z* 233 $[M+H]^+$.

- **Synthesis of TS3: (S,*E*)-3-(3,4-dihydroxyphenyl)-2-((4-oxopent-2-en-2-yl)amino)propanoate**

An ethanolic solution (5 mL) of (S)-methyl 2-amino-3-(3,4-dihydroxyphenyl)propanoate hydrochloride (0.30 g, 1.21 mmol), 0.12 g (1.21 mmol) of Et_3N and 0.12 g (1.21 mmol) of acetyl acetone was heated at reflux temperature for 41 hours monitored by TLC (DCM- EtOH 9.8:0.2). Once the reaction was completed the solvent was removed under reduced pressure and the residue was treated with DCM and washed with distilled water (3x 10 mL). The collected organic phase was dried on anhydrous $MgSO_4$, filtered and evaporated resulting in a chromatographically pure yellow solid.
MW: 293.32 (g/mol); **Yield**: 30%; **Mp**: 47-49 °C; **Rf**: 0.48 (DCM - EtOH 9.8 : 0.2); **IR**: (nujol, cm^{-1}): 3467 (NH), 1724 (C=O ester), 1606 (C=O ketone);
^{1}H-NMR ($CDCl_3$, δ, ppm): 10.99 (d, J = 8.6 Hz, 1H, NH), 7.97 (s, 1H, OH), 6.86 – 6.74 (m, 3H, arom. + OH), 6.58 (dd, J = 8.0, 2.2 Hz, 1H, arom.), 4.99 (s, 1H, CH), 4.33 (td, J = 8.0, 4.4 Hz, 1H, =CH-), 3.72 (s, 3H, OCH_3), 3.14 – 2.91 (m, 2H, CH_2), 2.01 (s, 3H, CH_3), 1.75 (s, 3H, CH_3); **^{13}C-NMR** ($CDCl_3$, δ, ppm): 195.90, 171.27, 163.75, 144.53, 144.15, 126.90, 120.98, 116.22, 115.22, 96.83, 96.76, 58.05, 52.65, 38.79, 28.15, 19.06. **MS**: *m/z* 294 $[M+H]^+$. Anal. calcd for $C_{15}H_{19}NO_5$ (%): C, 61.42; H, 6.53; N, 4.78. Found: C, 61.50; H, 6.30; N, 4.96.

In the same manner, but with different reaction times, compounds **TS4, TS5** and **TS6** were synthesized.

- **TS4: Methyl (S,*E*)-3-(4-(benzyloxy)phenyl)-2-((4-oxopent-2-en-2-yl)amino)propanoate**

Light orange solid; **MW**: 367.45 (g/mol); **Yield**: 42%; **Mp**: olio; **Rf**: 0.31 (DCM - EtOH 9.9 : 0.1); **IR**: (nujol, cm^{-1}): 3463 (NH), 1733 (C=O ester), 1622 (C=O ketone); **^{1}H-NMR** ($CDCl_3$, δ, ppm): 11.05 (d, J = 9.3 Hz, 1H, NH), 7.45 – 7.32 (m, 4H, arom.), 7.36 – 7.28 (m, 1H, arom.), 7.12 (d, J = 8.7 Hz, 2H, arom.), 6.90 (d, J = 6.6 Hz, 1H, arom.), 5.03 (s, 2H, CH_2-O), 4.93 (s, 1H, CH), 4.26 (td, J = 8.9, 4.8 Hz, 1H, =CH-), 3.72 (s, 3H, OCH_3), 3.18 – 3.04 (m, 1H, C*H*H), 2.94 (dd, J = 13.8, 8.8 Hz, 1H, CH*H*), 2.01 (s, 3H, CH_3), 1.61 (s, 3H, CH_3); **^{13}C-NMR** ($CDCl_3$, δ, ppm): 195.90, 171.48, 161.27, 157.92, 136.90, 130.40, 128.53, 128.34, 127.92, 127.48, 115.02, 96.66, 96.59, 69.95, 58.25, 39.10, 29.02, 18.78. **MS**: *m/z* 368 $[M+H]^+$. Anal. calcd for $C_{22}H_{25}NO_4$ (%): C, 71.91; H, 6.86; N, 3.81. Found: C, 71.90; H, 6.70; N, 3.91.

- **TS5: Methyl (S,*E*)-3-(4-hydroxyphenyl)-2-((4-oxopent-2-en-2-yl)amino)propanoate**

Yellow solid: **MW**: 277.32 (g/mol); **Yield**: 46%; Mp: 155-7 °C; **Rf**: 0.41 (DCM - EtOH 9.8 : 0.2); **IR**: (nujol, cm^{-1}): 3446 (NH),1743 (C=O ester), 1606 (C=O ketone);
^{1}H-NMR ($CDCl_3$, δ, ppm): 10.99 (d, J = 8.5 Hz, 1H, NH), 8.64 (s br, 1H, OH), 7.15 – 6.94 (m, 2H, arom.), 6.85 – 6.68 (m, 2H, arom.), 4.99 (s, 1H, CH), 4.36 (td, J = 8.4, 4.4 Hz, 1H, =CH-), 3.72 (s, 3H, OCH_3), 3.17 – 2.91 (m, 2H, CH_2), 2.02 (s, 3H, CH_3), 1.76 (s, 3H, CH_3); **^{13}C-NMR** ($CDCl_3$, δ, ppm): 196.09, 171.34, 162.79, 156.43, 130.37, 126.02, 115.79, 96.86, 96.76, 58.06, 52.64, 38.53, 28.35, 19.05. **MS**: *m/z* 278 $[M+H]^+$. Anal. calcd for $C_{15}H_{19}NO_4$ (%): C, 64.97; H, 6.91; N, 5.05. Found: C, 65.08; H, 6.80; N, 5.16.

- **TS6: Methyl (*E*)-3-(3,4-dihydroxyphenyl)-2-((4-oxopent-2-en-2-yl)amino) propanoate**

Yellow gummy solid; **MW**: 293.32 (g/mol); **Yield**: 51%; **Mp**: 42-44 °C; **Rf**: 0,41 (DCM - EtOH 9.6 : 0.4); **IR**: (nujol, cm^{-1}): 3467 (NH) 1723 (C=O ester), 1606 (C=O ketone); **^{1}H-NMR** ($CDCl_3$, δ, ppm): 11.00 (d, J = 8.8 Hz, 1H, NH), 6.84 – 6.72 (m, 2H, arom.), 6.58 (dd, J = 8.1, 2.1 Hz, 1H, arom.), 5.30 (s, 2H, 2xOH), 4.99 (s, 1H, CH), 4.34 (td, J = 8.4, 4.4 Hz, 1H, =CH-), 3.72 (s, 3H, OCH_3), 3.15 – 2.88 (m, 2H, CH_2), 2.01 (s, 3H, CH_3), 1.75 (s, 3H, CH_3); **^{13}C-NMR** ($CDCl_3$, δ, ppm): 195.87, 171.26, 163.58, 144.51, 144.25, 126.88, 120.94, 116.27, 115.13, 96.71, 58.04, 38.74, 19.06. **MS**: *m/z* 294 $[M+H]^+$. Anal. calcd for $C_{15}H_{19}NO_5$ (%): C, 61.42; H, 6.53; N, 4.78. Found: C, 61.62; H, 6.45; N, 4.70.
