# Supplementary material for: Wet-dry-wet drug screen leads to the synthesis of TS1, a novel compound reversing lung fibrosis through inhibition of myofibroblast differentiation
Source: Cell Death Dis. 2021 Dec 17;13(1):2. doi: 10.1038/s41419-021-04439-4 (PMC8677786; doi:10.1038/s41419-021-04439-4)
Supplement: Supplementary file 3 — Confirmation from all authors [file 41419_2021_4439_MOESM3_ESM.pdf]

# Email to all authors on 25.11.2021 at 10.10

URGENT RESPONSE NEEDED for publication!!
15 messages

Nadja Ring <nadjaring@gmail.com> 25 November 2021 at 10:10
To: Maria Concetta Volpe <maria.volpe@icgeb.org>, Tomaž Stepišnik <tomaz.stepisnik@ijs.si>, Maria Grazia Mamolo <mamolo@units.it>, Pance Panov <Pance.Panov@ijs.si>, Dragi Kocev <dragi.kocev@ijs.si>, Simone Vodret <Simone.Vodret@icgeb.org>, Sara Fortuna <s.fortuna@units.it>, Daniele Zampieri <dzampieri@units.it>, Francesco Loffredo Icgeb <Francesco.Loffredo@icgeb.org>, Saso Dzeroski <saso.dzeroski@ijs.si>, Serena Zacchigna <Serena.Zacchigna@icgeb.org>, Confalonieri Marco - ASUGI <marco.confalonieri@asugi.sanita.fvg.it>, Marco Confalonieri <mconfalonieri@units.it>, "Ligresti, Giovanni" <ligresti@bu.edu>, Michael Rehman <michael.rehman@outlook.com>, Michael Rehman <smirrims@live.com>, Andrea Colliva <Andrea.Colliva@icgeb.org>, Scarabellotto Sara <sara.scarabellotto@studenti.units.it>, "Caporarello, Nunzia" <Caporarello.Nunzia@mayo.edu>, Luca Camparini <Luca.Camparini@icgeb.org>, "Pham, Tho X" <txpham@bu.edu>, marchesan@icgeb.org, Thomas Marcuzzo <marcuzzothomas@gmail.com>, BUSSANI ROSSANA <bussani@units.it>, ANTONELLA.CALABRETTI@deams.units.it

Dear all,
We are close to finally publishing our work on TS1 in lung fibrosis in the Journal Cell Death and Disease. During the revision we received a lot of help from a new student of Serena's - Pietro Marchesan. He has therefore been added to the author list (I have pasted the complete author list below for your reference).
The journal requires an **EMAIL FROM ALL AUTHORS** confirming that you agree that we add this author. Please send this to me ASAP.

You can copy and paste this statement (or write your own) and send it to me with your email signature:

I am an author of the work titled "Wet-dry-wet drug screen leads to the synthesis of TS1, a novel compound reversing lung fibrosis through inhibition of myofibroblast differentiation". I am aware of, and agree with the addition of the author Pietro Marchesan to the author list.

Thank you everyone!
Best regards,
Nadja

Full author list:

Nadja Anneliese Ruth Ring1*, Maria Concetta Volpe1,2*, Tomaž Stepišnik3, Maria Grazia Mamolo4, Panče Panov3, Dragi Kocev3, Simone Vodret1, Sara Fortuna4, Antonella Calabretti4, Michael Rehman1,5, Andrea Colliva1, Pietro Marchesan1, Luca Camparini6, Thomas Marcuzzo2, Rossana Bussani2, Sara Scarabellotto4, Marco Confalonieri2, Tho X. Pham7, Giovanni Ligresti7, Nunzia Caporarello8, Francesco S. Loffredo6,9, Daniele Zampieri4, Sašo Džeroski3 and Serena Zacchigna1,2

Responses in order of authorship:

Nadja Anneliese Ruth Ring

agreement with new author
1 message

Nadja Ring <nadja.ring@icgeb.org> 25 November 2021 at 11:23
To: "...me" <nadjaring@gmail.com>

I am an author of the work titled "Wet-dry-wet drug screen leads to the synthesis of TS1, a novel compound reversing lung fibrosis through inhibition of myofibroblast differentiation". I am aware of, and agree with the addition of the author Pietro Marchesan to the author list.

Kind regards,
Dr. Nadja Ring

Maria Concetta Volpe

Maria VOLPE <Maria.Volpe@icgeb.org> 25 November 2021 at 11:25
To: Nadja Ring <nadjaring@gmail.com>
Cc: Maria VOLPE <Maria.Volpe@icgeb.org>, Tomaž Stepišnik <tomaz.stepisnik@ijs.si>, Maria Grazia Mamolo <mamolo@units.it>, Pance Panov <Pance.Panov@ijs.si>, Dragi Kocev <dragi.kocev@ijs.si>, Simone VODRET <Simone.Vodret@icgeb.org>, Sara Fortuna <s.fortuna@units.it>, Daniele Zampieri <dzampieri@units.it>, Francesco LOFFREDO <Francesco.Loffredo@icgeb.org>, Saso Dzeroski <saso.dzeroski@ijs.si>, Serena ZACCHIGNA <Serena.Zacchigna@icgeb.org>, Confalonieri Marco - ASUGI <marco.confalonieri@asugi.sanita.fvg.it>, Marco Confalonieri <mconfalonieri@units.it>, "Ligresti, Giovanni" <ligresti@bu.edu>, Michael Rehman <michael.rehman@outlook.com>, Michael Rehman <smirrims@live.com>, Andrea COLLIVA <Andrea.Colliva@icgeb.org>, Scarabellotto Sara <sara.scarabellotto@studenti.units.it>, "Caporarello, Nunzia" <Caporarello.Nunzia@mayo.edu>, Luca CAMPARINI <Luca.Camparini@icgeb.org>, "Pham, Tho X" <txpham@bu.edu>, "marchesan@icgeb.org" <marchesan@icgeb.org>, Thomas Marcuzzo <marcuzzothomas@gmail.com>, BUSSANI ROSSANA <bussani@units.it>, "ANTONELLA.CALABRETTI@deams.units.it" <ANTONELLA.CALABRETTI@deams.units.it>

I am an author of the work titled "Wet-dry-wet drug screen leads to the synthesis of TS1, a novel compound reversing lung fibrosis through inhibition of myofibroblast differentiation". I am aware of, and agree with the addition of the author Pietro Marchesan to the author list.

MV

Il giorno 25 nov 2021, alle ore 10:10, Nadja Ring <nadjaring@gmail.com> ha scritto:

[Quoted text hidden]

Tomaž Stepišnik

Tomaž Stepišnik <tomaz.stepi@gmail.com> 25 November 2021 at 11:11
To: Nadja Ring <nadjaring@gmail.com>

I confirm that I am an author of the work titled "Wet-dry-wet drug screen leads to the synthesis of TS1, a novel compound reversing lung fibrosis through inhibition of myofibroblast differentiation". I agree with the addition of the author Pietro Marchesan to the author list.

Best regards,
Tomaž Stepišnik
[Quoted text hidden]

Maria Grazia Mamolo

MAMOLO MARIA GRAZIA <mamolo@units.it> 25 November 2021 at 10:57
To: Nadja Ring <nadjaring@gmail.com>, Maria Concetta Volpe <maria.volpe@icgeb.org>, Tomaž Stepišnik <tomaz.stepisnik@ijs.si>, Pance Panov <Pance.Panov@ijs.si>, Dragi Kocev <dragi.kocev@ijs.si>, Simone Vodret <Simone.Vodret@icgeb.org>, FORTUNA SARA <s.fortuna@units.it>, ZAMPIERI DANIELE <dzampieri@units.it>, Francesco Loffredo Icgeb <Francesco.Loffredo@icgeb.org>, Saso Dzeroski <saso.dzeroski@ijs.si>, Serena Zacchigna <Serena.Zacchigna@icgeb.org>, Confalonieri Marco - ASUGI <marco.confalonieri@asugi.sanita.fvg.it>, CONFALONIERI MARCO <mconfalonieri@units.it>, "Ligresti, Giovanni" <ligresti@bu.edu>, Michael Rehman <michael.rehman@outlook.com>, Michael Rehman <smirrims@live.com>, Andrea Colliva <Andrea.Colliva@icgeb.org>, "SCARABELLOTTO SARA [FA0200193]" <SARA.SCARABELLOTTO@studenti.units.it>, "Caporarello, Nunzia" <Caporarello.Nunzia@mayo.edu>, Luca Camparini <Luca.Camparini@icgeb.org>, "Pham, Tho X" <txpham@bu.edu>, "marchesan@icgeb.org" <marchesan@icgeb.org>, Thomas Marcuzzo <marcuzzothomas@gmail.com>, BUSSANI ROSSANA <bussani@units.it>, CALABRETTI ANTONELLA <ANTONELLA.CALABRETTI@deams.units.it>

I am an author of the work titled "Wet-dry-wet drug screen leads to the synthesis of TS1, a novel compound reversing lung fibrosis through inhibition of myofibroblast differentiation". I am aware of, and agree with the addition of the author Pietro Marchesan to the author list.

Maria Grazia Mamolo
Associate Professor Medicinal Chemistry
Department of Chemistry and Pharmaceutical Sciences
University of Trieste Italy

Panče Panov

URGENT RESPONSE NEEDED for publication!!

Pance Panov <pance.panov@ijs.si> 25 November 2021 at 11:59
Reply-To: pance.panov@ijs.si
To: Nadja Ring <nadjaring@gmail.com>

I am an author of the work titled "Wet-dry-wet drug screen leads to the synthesis of TS1, a novel compound reversing lung fibrosis through inhibition of myofibroblast differentiation". I am aware of, and agree with the addition of the author Pietro Marchesan to the author list.

Panče Panov
Jožef Stefan Institute
Ljubljana, Slovenia
[Quoted text hidden]
--
dr. Panče Panov
Research Associate / Assistant Professor

Jožef Stefan Institute
Department of Knowledge Technologies
Jamova cesta 39
SI-1000 Ljubljana Slovenia

Contact information
Office at Teslova 30, second floor, room 70A
Office phone: +386 1 477 3016
Mobile phone: +386 40 487 388
Web page: http://kt.ijs.si/panovp/

Dragi Kocev

Our work on TS1 in lung fibrosis in the Journal Cell Death and Disease
1 message

Dragi Kocev <Dragi.Kocev@ijs.si> 25 November 2021 at 12:23
To: Nadja Ring <nadjaring@gmail.com>

Dear Nadja,

I am an author of the work titled "Wet-dry-wet drug screen leads to the synthesis of TS1, a novel compound reversing lung fibrosis through inhibition of myofibroblast differentiation". I am aware of, and agree with the addition of the author Pietro Marchesan to the author list.

With best regards,

Dragi Kocev

--
Department of Knowledge Technologies
Jozef Stefan Institute, Ljubljana, Slovenia
URL: http://kt.ijs.si/DragiKocev/

Simone Vodret

Simone Vodret <Simone.Vodret@icgeb.org> 25 November 2021 at 10:36
To: Nadja Ring <nadjaring@gmail.com>

Ciao Nadja!!!!!!!!!!!

See the attachment

Trieste, 25/11/2021

I am an author of the work titled "Wet-dry-wet drug screen leads to the synthesis of TS1, a novel compound reversing lung fibrosis through inhibition of myofibroblast differentiation". I am aware of, and agree with the addition of the author Pietro Marchesan to the author list.

Best regards

Simone Vodret

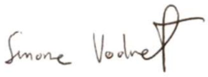

Sara Fortuna

Statement
1 message

FORTUNA SARA <s.fortuna@units.it> 25 November 2021 at 11:28
To: "nadja.ring@icgeb.org" <nadja.ring@icgeb.org>

I am an author of the work titled "Wet-dry-wet drug screen leads to the synthesis of TS1, a novel compound reversing lung fibrosis through inhibition of myofibroblast differentiation". I am aware of, and agree with the addition of the author Pietro Marchesan to the author list.

Sara Fortuna

Università degli Studi di Trieste | University of Trieste
Dipartimento di Scienze Chimiche e Farmaceutiche (DSCF) | Department of Chemical and Pharmaceutical Sciences

Via L. Giorgieri, 1 - 34127 Trieste (Italy)

s.fortuna@units.it - www.sarafortuna.eu
Tel.| Ph. +39 040 558 3671
Skype: sara78_

Antonella Calabretti

URGENT RESPONSE NEEDED for publication!!

CALABRETTI ANTONELLA <ANTONELLA.CALABRETTI@deams.units.it> 25 November 2021 at 18:23
To: Nadja Ring <nadjaring@gmail.com>

I am an author of the work titled "Wet-dry-wet drug screen leads to the synthesis of TS1, a novel compound reversing lung fibrosis through inhibition of myofibroblast differentiation". I am aware of, and agree with the addition of the author Pietro Marchesan to the author list.

Antonella Calabretti,
Laboratorio Merceologico
DEAMS - Università degli Studi di Trieste
Via A. Valerio, 6, 34127 Trieste
Tel. 040 5587084
Cell +39 393 8084221

Michael Rehman

URGENT RESPONSE NEEDED for publication!!

Michael Rehman <michael.rehman@outlook.com> 25 November 2021 at 16:16
To: Nadja Ring <nadjaring@gmail.com>
Cc: Maria Concetta Volpe <Maria.Volpe@icgeb.org>, Tomaž Stepišnik <tomaz.stepisnik@ijs.si>, Maria Grazia Mamolo <mamolo@units.it>, Pance Panov <Pance.Panov@ijs.si>, Dragi Kocev <dragi.kocev@ijs.si>, Simone Vodret <Simone.Vodret@icgeb.org>, Sara Fortuna <s.fortuna@units.it>, Daniele Zampieri <dzampieri@units.it>, Francesco Loffredo Icgeb <Francesco.Loffredo@icgeb.org>, Saso Dzeroski <saso.dzeroski@ijs.si>, Serena Zacchigna <Serena.Zacchigna@icgeb.org>, Confalonieri Marco - ASUGI <marco.confalonieri@asugi.sanita.fvg.it>, Marco Confalonieri <mconfalonieri@units.it>, "Ligresti, Giovanni" <ligresti@bu.edu>, Michael Rehman <smirrims@live.com>, Andrea Colliva <Andrea.Colliva@icgeb.org>, Scarabellotto Sara <sara.scarabellotto@studenti.units.it>, "Caporarello, Nunzia" <Caporarello.Nunzia@mayo.edu>, Luca Camparini <Luca.Camparini@icgeb.org>, "Pham, Tho X" <txpham@bu.edu>, "marchesan@icgeb.org" <marchesan@icgeb.org>, BUSSANI ROSSANA <bussani@units.it>, "ANTONELLA.CALABRETTI@deams.units.it" <ANTONELLA.CALABRETTI@deams.units.it>, Thomas Marcuzzo <marcuzzothomas@gmail.com>

I am an author of the work titled "Wet-dry-wet drug screen leads to the synthesis of TS1, a novel compound reversing lung fibrosis through inhibition of myofibroblast differentiation". I am aware of, and agree with the addition of the author Pietro Marchesan to the author list.

Michael Rehman
Associate Research Scientist
Yale School of Medicine
Section of Nephrology
300 Cedar Street, TAC S360
New haven, CT 06520, USA

Andrea Colliva

Author list
1 message

Andrea COLLIVA <Andrea.Colliva@icgeb.org> 25 November 2021 at 11:22
To: Nadja Ring <nadjaring@gmail.com>

I am an author of the work titled "Wet-dry-wet drug screen leads to the synthesis of TS1, a novel compound reversing lung fibrosis through inhibition of myofibroblast differentiation". I am aware of, and agree with the addition of the author Pietro Marchesan to the author list.

Andrea Colliva, PhD

Cardiovascular Biology lab

ICGEB Trieste, Italy

Padriciano 99, 34149 Trieste

Tel. 040-3757214

Email: andrea.colliva@icgeb.org

Pietro Marchesan

URGENT RESPONSE NEEDED for publication!!

MARCHESAN PIETRO [ME0200385] <PIETRO.MARCHESAN@studenti.units.it> 25 November 2021 at 18:10
To: Nadja Ring <nadjaring@gmail.com>

I am an author of the work titled "Wet-dry-wet drug screen leads to the synthesis of TS1, a novel compound reversing lung fibrosis through inhibition of myofibroblast differentiation". I am aware of, and agree with the addition of the author Pietro Marchesan to the author list.

Best regards,

Pietro Marchesan

Luca Camparini

URGENT RESPONSE NEEDED for publication!!

Luca Camparini <luca.camparini.1@gmail.com> 25 November 2021 at 17:55
To: Nadja Ring <nadjaring@gmail.com>

Dear Nadja,

Thank you for your message and for the good news. As you requested I can confirm that:

I am an author of the work titled "Wet-dry-wet drug screen leads to the synthesis of TS1, a novel compound reversing lung fibrosis through inhibition of myofibroblast differentiation". I am aware of, and agree with the addition of the author Pietro Marchesan to the author list.

Let me know if you need something else.
Best regards

Luca Camparini

Thomas Marcuzzo

URGENT RESPONSE NEEDED for publication!!

Thomas Marcuzzo <marcuzzothomas@gmail.com> 25 November 2021 at 11:49
To: Nadja Ring <nadjaring@gmail.com>
Cc: Maria Concetta Volpe <Maria.Volpe@icgeb.org>, Tomaž Stepišnik <tomaz.stepisnik@ijs.si>, Maria Grazia Mamolo <mamolo@units.it>, Pance Panov <Pance.Panov@ijs.si>, Dragi Kocev <dragi.kocev@ijs.si>, Simone Vodret <Simone.Vodret@icgeb.org>, Sara Fortuna <s.fortuna@units.it>, Daniele Zampieri <dzampieri@units.it>, Francesco Loffredo Icgeb <Francesco.Loffredo@icgeb.org>, Saso Dzeroski <saso.dzeroski@ijs.si>, Serena Zacchigna <Serena.Zacchigna@icgeb.org>, Confalonieri Marco - ASUGI <marco.confalonieri@asugi.sanita.fvg.it>, Marco Confalonieri <mconfalonieri@units.it>, "Ligresti, Giovanni" <ligresti@bu.edu>, Michael Rehman <michael.rehman@outlook.com>, Michael Rehman <smirrims@live.com>, Andrea Colliva <Andrea.Colliva@icgeb.org>, Scarabellotto Sara <sara.scarabellotto@studenti.units.it>, "Caporarello, Nunzia" <Caporarello.Nunzia@mayo.edu>, Luca Camparini <Luca.Camparini@icgeb.org>, "Pham, Tho X" <txpham@bu.edu>, marchesan@icgeb.org, BUSSANI ROSSANA <bussani@units.it>, ANTONELLA.CALABRETTI@deams.units.it

I am an author of the work titled "Wet-dry-wet drug screen leads to the synthesis of TS1, a novel compound reversing lung fibrosis through inhibition of myofibroblast differentiation". I am aware of, and agree with the addition of the author Pietro Marchesan to the author list.

Thank you
Thomas Marcuzzo
[Quoted text hidden]

Rossana Bussani

Confirm my partecipation as author.
1 message

Bussani Rossana - ASUGI <rossana.bussani@asugi.sanita.fvg.it> 25 November 2021 at 11:14
To: "nadjaring@gmail.com" <nadjaring@gmail.com>

I am an author of the work titled "Wet-dry-wet drug screen leads to the synthesis of TS1, a novel compound reversing lung fibrosis through inhibition of myofibroblast differentiation". I am aware of, and agree with the addition of the author Pietro Marchesan to the author list.

Best regards,

rossana bussani.

Sara Scarabellotto

URGENT RESPONSE NEEDED for publication!!

Sara Scarabellotto <sscarabellotto94@gmail.com> 25 November 2021 at 16:31
To: Nadja Ring <nadjaring@gmail.com>

I am an author of the work titled "Wet-dry-wet drug screen leads to the synthesis of TS1, a novel compound reversing lung fibrosis through inhibition of myofibroblast differentiation". I am aware of, and agree with the addition of the author Pietro Marchesan to the author list.

Sara Scarabellotto
[Quoted text hidden]

Marco Confalonieri

Confalonieri Marco - ASUGI <marco.confalonieri@asugi.sanita.fvg.it> 25 November 2021 at 10:58
To: MAMOLO MARIA GRAZIA <mamolo@units.it>, Nadja Ring <nadjaring@gmail.com>, Maria Concetta Volpe <maria.volpe@icgeb.org>, Tomaž Stepišnik <tomaz.stepisnik@ijs.si>, Pance Panov <Pance.Panov@ijs.si>, Dragi Kocev <dragi.kocev@ijs.si>, Simone Vodret <Simone.Vodret@icgeb.org>, FORTUNA SARA <s.fortuna@units.it>, ZAMPIERI DANIELE <dzampieri@units.it>, Francesco Loffredo Icgeb <Francesco.Loffredo@icgeb.org>, Saso Dzeroski <saso.dzeroski@ijs.si>, Serena Zacchigna <Serena.Zacchigna@icgeb.org>, CONFALONIERI MARCO <mconfalonieri@units.it>, "Ligresti, Giovanni" <ligresti@bu.edu>, Michael Rehman <michael.rehman@outlook.com>, Michael Rehman <smirrims@live.com>, Andrea Colliva <Andrea.Colliva@icgeb.org>, "SCARABELLOTTO SARA [FA0200193]" <SARA.SCARABELLOTTO@studenti.units.it>, "Caporarello, Nunzia" <Caporarello.Nunzia@mayo.edu>, Luca Camparini <Luca.Camparini@icgeb.org>, "Pham, Tho X" <txpham@bu.edu>, "marchesan@icgeb.org" <marchesan@icgeb.org>, Thomas Marcuzzo <marcuzzothomas@gmail.com>, BUSSANI ROSSANA <bussani@units.it>, CALABRETTI ANTONELLA <ANTONELLA.CALABRETTI@deams.units.it>

I also agree

Marco Confalonieri

Tho X. Pham

URGENT RESPONSE NEEDED for publication!!

Pham, Tho X <txpham@bu.edu> 25 November 2021 at 14:15
To: Nadja Ring <nadjaring@gmail.com>

I am an author of the work titled "Wet-dry-wet drug screen leads to the synthesis of TS1, a novel compound reversing lung fibrosis through inhibition of myofibroblast differentiation". I am aware of, and agree with the addition of the author Pietro Marchesan to the author list.

Best,
Tho

Giovanni Ligresti

URGENT RESPONSE NEEDED for publication!!

Ligresti, Giovanni <ligresti@bu.edu> 26 November 2021 at 14:23
To: Nadja Ring <nadjaring@gmail.com>

I am an author of the work titled "Wet-dry-wet drug screen leads to the synthesis of TS1, a novel compound reversing lung fibrosis through inhibition of myofibroblast differentiation". I am aware of, and agree with the addition of the author Pietro Marchesan to the author list.

Best,

Giovanni

Giovanni Ligresti, PhD

Assistant Professor of Medicine

Department of Medicine

Arthritis Center/Pulmonary Center

Boston University School of Medicine

Email: ligresti@bu.edu

Phone: 617-358-6786

---

72 East Concord St., E-5

Boston, MA 02118

Nunzia Caporarello

URGENT RESPONSE NEEDED for publication!!

Caporarello, Nunzia, Ph.D. <Caporarello.Nunzia@mayo.edu> 25 November 2021 at 17:49
To: Nadja Ring <nadjaring@gmail.com>, Maria Concetta Volpe <maria.volpe@icgeb.org>, Tomaž Stepišnik <tomaz.stepisnik@ijs.si>, Maria Grazia Mamolo <mamolo@units.it>, Pance Panov <Pance.Panov@ijs.si>, Dragi Kocev <dragi.kocev@ijs.si>, Simone Vodret <Simone.Vodret@icgeb.org>, Sara Fortuna <s.fortuna@units.it>, Daniele Zampieri <dzampieri@units.it>, Francesco Loffredo Icgeb <Francesco.Loffredo@icgeb.org>, Saso Dzeroski <saso.dzeroski@ijs.si>, Serena Zacchigna <Serena.Zacchigna@icgeb.org>, Confalonieri Marco - ASUGI <marco.confalonieri@asugi.sanita.fvg.it>, Marco Confalonieri <mconfalonieri@units.it>, "Ligresti, Giovanni" <ligresti@bu.edu>, Michael Rehman <michael.rehman@outlook.com>, Michael Rehman <smirrims@live.com>, Andrea Colliva <Andrea.Colliva@icgeb.org>, Scarabellotto Sara <sara.scarabellotto@studenti.units.it>, Luca Camparini <Luca.Camparini@icgeb.org>, "Pham, Tho X" <txpham@bu.edu>, "marchesan@icgeb.org" <marchesan@icgeb.org>, Thomas Marcuzzo <marcuzzothomas@gmail.com>, BUSSANI ROSSANA <bussani@units.it>, "ANTONELLA.CALABRETTI@deams.units.it" <ANTONELLA.CALABRETTI@deams.units.it>

I am an author of the work titled "Wet-dry-wet drug screen leads to the synthesis of TS1, a novel compound reversing lung fibrosis through inhibition of myofibroblast differentiation". I am aware of, and agree with the addition of the author Pietro Marchesan to the author list.

Nunzia Caporarello, Ph.D.

Assistant Professor of Physiology

Department of Physiology & Biomedical Engineering

Mayo Clinic College of Medicine and Science
Phone: 507-255-8475
Email: caporarello.nunzia@mayo.edu

Mayo Clinic
200 First Street SW Rochester, MN 55905
mayoclinic.org

Francesco S. Loffredo

Francesco Loffredo <loffredo@icgeb.org> 25 November 2021 at 10:52
To: Nadja Ring <nadjaring@gmail.com>

I am an author of the work titled "Wet-dry-wet drug screen leads to the synthesis of TS1, a novel compound reversing lung fibrosis through inhibition of myofibroblast differentiation". I am aware of, and agree with the addition of the author Pietro Marchesan to the author list.
Regards

Francesco Loffredo

Francesco Loffredo MD, PhD
Head of Molecular Cardiology
International Centre for Genetic
Engineering and Biotechnology
34149 Trieste, Italy

Associate Professor of Cardiology
Department of Translational Medical Sciences
University of Campania "Luigi Vanvitelli"
Vanvitelli Cardiology and Cardiac Intensive Care Unit
Monaldi Hospital 80131 Naples, Italy

[Quoted text hidden]

Daniele Zampieri

Agreement
1 message

Daniele Zampieri <dzampieri@units.it> 25 November 2021 at 10:57
To: Nadja Ring <nadjaring@gmail.com>

I am an author of the work titled "Wet-dry-wet drug screen leads to the synthesis of TS1, a novel compound reversing lung fibrosis through inhibition of myofibroblast differentiation". I am aware of, and agree with the addition of the author Pietro Marchesan to the author list.

Sincerely,

Danierle Zampieri

Daniele Zampieri
Dr. | Ph.D. Assistant Professor
Università degli Studi di Trieste | University of Trieste
Dipartimento di Scienze Chimiche e Farmaceutiche (DSCF) |
Department of Chemical and Pharmaceutical Sciences
Via L. Giorgieri, 1 - 34127 Trieste (Italy)
dzampieri@units.it
Tel.| Ph. Office: +39 040 558-7858; labs: -3695 / -3686
Cell. | Mob. +39 3394914314
Skype: d.zampieri7

Sašo Džeroski

Saso Dzeroski <Saso.Dzeroski@ijs.si> 25 November 2021 at 11:00
To: Nadja Ring <nadjaring@gmail.com>

I am an author of the work titled "Wet-dry-wet drug screen leads to the synthesis of TS1, a novel compound reversing lung fibrosis through inhibition of myofibroblast differentiation". I am aware of, and agree with the addition of the author Pietro Marchesan to the author list.

Professor Saso Dzeroski

Head of Department of Knowledge technologies

Jozef Stefan Institute, Jamova cesta 39, Ljubljana, Slovenia

>>-->

[Quoted text hidden]
